# Analysis of the genomes of a male-killing Spiroplasma and its co-infecting Rickettsia reveals a case of concerted genome expansion

**DOI:** 10.1099/mgen.0.001766

**Published:** 2026-07-16

**Authors:** Emily A. Hornett, Masayuki Hayashi, Keisuke Nagamine, Steve Paterson, Daisuke Kageyama, Gregory D. D. Hurst

**Affiliations:** 1 Department of Evolution, Ecology & Behaviour, University of Liverpool, Liverpool, UK; 2 Department of Vector Biology, Liverpool School of Tropical Medicine, Liverpool, UK; 3 Institute for Plant Protection, National Agriculture and Food Research Organization, Tsukuba, Japan; 4 Institute of Agrobiological Sciences, National Agriculture and Food Research Organization, Tsukuba, Japan

**Keywords:** genome size, male-killing, mobile genetic elements, *Rickettsia*, *Spiroplasma*, symbiont

## Abstract

Maternally inherited symbionts are central to arthropod biology, functioning both as mutualistic partners and as reproductive parasites. Genomic analyses provide critical insight into these interactions. Here, we sequenced, assembled and examined the genomes of *Spiroplasma* and *Rickettsia* co-infecting the lacewing *Mallada desjardinsi*, with the aim of elucidating the male-killing phenotype of *Spiroplasma* and predicting potential phenotypes for *Rickettsia*. In *Spiroplasma*, we identified a set of candidate effector genes. However, Spaid-like genes, where present, lacked the functional domains previously demonstrated to be important for male-killing. The *Rickettsia* genome contained two *cifA/cifB* gene pairs, consistent with the capacity to induce cytoplasmic incompatibility (CI). Unexpectedly, both symbiont genomes were markedly expanded relative to congenerics. We describe this pattern – that contrasts with the classical trajectory of genome reduction in symbionts – as secondary genome expansion. Expansion was driven by extensive proliferation of mobile elements: *Rickettsia* harboured an exceptionally high number of insertion sequences, while *Spiroplasma* accumulated both insertion sequences and prophage regions. Collectively, our findings indicate that the canonical Spaid-mediated male-killing mechanism is not conserved in the *Spiroplasma* of *M. desjardinsi*, while *Rickettsia* may induce CI. Moreover, the parallel genome expansions observed suggest that secondary expansion events may be influenced by host-associated factors rather than occurring stochastically.

**Impact Statement**

Many insects carry heritable symbionts – microbes that pass from a female host to her progeny – that impact host biology. Sequencing the genomes of these microbes is foundational to understanding how they function as symbionts and our understanding of how symbionts evolve. We sequenced two bacterial symbionts – a *Spiroplasma* and *Rickettsia* – that coinfect a lacewing insect, driven by a desire to understand the enigmatic symbiont trait of male-killing, where the symbiont selectively kills male hosts. The *Spiroplasma* from the lacewing did not carry functional copies of a known male-killing gene from *Spiroplasma* infecting flies (Spaid), suggesting diverse male-killing mechanisms within the genus. It did carry other predicted virulence determinants in the form of a gene with an ETX/MTX2 toxin domain alongside a second gene with a ribosome-inactivating protein domain, presenting new candidates for the male-killing process. The *Rickettsia* carried elements known to underpin cytoplasmic incompatibility. The most remarkable observation was unexpected – the size of the symbiont genomes. Symbiont genomes are normally streamlined and small in size. These two symbionts, however, both had greatly expanded genomes, each the largest in their respective groups. For both symbionts, the genomes were highly repetitive, and the increase in genome size was fuelled by overproliferation of mobile genetic elements. This observation of concerted genome expansion suggests that secondary expansion events in symbionts may not be random but could be driven by their host’s biology.

## Data Summary

The sequence read data (SAMN49005244) and symbiont genomes (SAMN49005778 and SAMN49005944) are publicly available in NCBI under the BioProject ID PRJNA1274929.

## Introduction

Arthropod biology, ecology and evolution are driven in part by the symbioses they form with microbes [1]. In many cases, these microbes are vertically transmitted, being passed from a female to her progeny on or in eggs. Heritable symbionts such as these are particularly significant, as their transmission relies on the survival and reproduction of their host, such that they have been selected to contribute to host physiology and function in diverse ways [2,4]. Conversely, their exclusively maternal inheritance selects for a variety of sex ratio distorting phenotypes that promote the production or survival of female offspring [5]. One of the most broadly found of these reproductive parasitic traits is male-killing, where heritable microbes kill male embryos that carry them. This trait has evolved in diverse bacterial symbionts, including *Wolbachia*, *Rickettsia*, *Spiroplasma* and *Arsenophonus*, as well as vertically transmitted viruses [6].

Understanding the genetic basis of symbiosis, and of symbiont phenotypes such as male-killing, is commonly first approached through examining the genome sequence of the symbionts. These genomes then generate hypotheses as to function in two ways. First, through comparison to known functional elements. The presence of a eukaryote-like association module in *Wolbachia* phage led to the hypothesis of *cifA/cifB* gene involvement in the phenotype of cytoplasmic incompatibility [7], later validated through functional genetics [8,10]. With the identification of these factors, the capacity of uncharacterized *Wolbachia* to induce CI could then be inferred, and further analysis indicated the breadth of taxa in which incompatibility induced by *cif* genes was occurring in other symbionts [11,12]. Second, through a comparative genomics approach, the genomes of related symbionts with different phenotypes can be contrasted to generate hypotheses as to the functional basis of traits. For instance, comparison of closely related male-killing and non-male-killing strains of *Wolbachia* in the tea tortrix moth revealed that the strains were nearly identical, save for a prophage element only present in the male-killing strain, which contained the gene *Oscar* [13]. This gene was later shown to underpin male-killing through interference with dosage compensation [14,15]. In a similar manner, the presence of prophage APSE in isolates of *Hamiltonella defensa* that defended pea aphids against wasp attack, and its absence in non-protective strains, implicated the genes within the prophage in the protective phenotype [16].

Our focal system is the interaction of the lacewing *Mallada desjardinsi* with its two heritable symbionts. The first symbiont is a member of the bacterial genus *Spiroplasma*, which includes insect pathogens, insect-vectored plant pathogens and heritable symbionts of insects [17]. The strain in *M. desjardinsi* is heritable, is present in 73.5% of female lacewings and expresses a male-killing phenotype where infected male hosts are killed during embryogenesis or early larval stages [18]. This male-killer is of particular interest, as the host species also carries a suppressor of male-killing that restores male host survival, and the system affords the opportunity to reconcile the male-killing mechanism with the host’s evolutionary response [19]. Male-killing *Spiroplasma* have been recorded in diverse other species, including *Drosophila*, ladybirds, aphids and lepidopterans, and phylogenetic analysis indicates that there are several independent evolutionary transitions in the genus *Spiroplasma* to the male-killing phenotype [20,24]. The genetic basis of male-killing by *Spiroplasma* has been determined in one case. The Spaid toxin protein comprises a signal peptide, ankyrin domain repeats, an Ovarian Tumor (OTU) deubiquitinase domain and a C-terminal hydrophobic region [25]. Spaid male-killing activity is dependent on both the ankyrin and OTU domains, and the toxin impacts the X chromosome in males which is sensitive by virtue of expressing a functional dosage compensation complex [26,27]. The absence of Spaid genes in male-killing *Spiroplasma* from aphids and the tea tortrix moth implies that male-killing has multiple causal bases in the symbiont clade [28,29]. *Spiroplasma* may also carry RIP (ribosome-inactivating protein) genes that function in defence against natural enemies [30]. Indeed, the *Spiroplasma* in *Drosophila melanogaster* encodes both Spaid that induces male-killing, and RIP toxins, which protect the fly against wasp attack [31]. In one case, *Spiroplasma* can induce cytoplasmic incompatibility [32].

The second symbiont that is present in *M. desjardinsi* is a member of the bacterial genus *Rickettsia. Rickettsia* are obligate host-associated intracellular microbes. Members of the genus vary from arthropod heritable symbionts to arthropod-vectored pathogens [33]. The *Rickettsia* in *M. desjardinsi* lies in the bellii group, first characterized in the form of *R. bellii* strains that are tick-associated heritable symbionts, and which is now known to also include a range of insect symbionts [34,35]. The impact of *Rickettsia* on the lacewing host is not known, aside from not being associated with sex ratio distortion. It is present in both male and female hosts, and surveys indicate that 73.5% of the population carry the symbiont. *Rickettsia* are known to cause cytoplasmic incompatibility in certain cases, underpinned by the *cifA/cifB* system [36], and may also be protective [37], but the mechanism underpinning protection has not been elucidated.

To enable insight into *Spiroplasma* and *Rickettsia* function, and onward study of the male-killer host interaction, we completed the genome sequence for both symbionts. We made use of an inbred line of *M. desjardinsi* that carried both *Spiroplasma* and *Rickettsia* as a coinfection. Our approach was metagenomic, using PacBio technology to sequence infected *M. desjardinsi*, then extracting the *Spiroplasma* and *Rickettsia* genomes from this pool.

## Methods

### DNA isolation and long-read sequencing

A single female *M. desjardinsi* lacewing was selected from a sixth-generation inbred line originating from Matsudo, Japan. This line was co-infected with two strains of bacteria: a *Spiroplasma* and a *Rickettsia*. To prepare the lacewing tissue for DNA isolation, the whole adult insect was first snap-frozen in liquid nitrogen, and the tissue was ground immediately using a sterile pestle. High-molecular-weight DNA was then isolated using a Qiagen Genomic-tip 20/G and associated buffers following the manufacturer's protocol (https://www.qiagen.com/). The resultant DNA was sequenced on a PacBio Sequel II SMRT Cell (HiFi) in CCS run mode (https://www.pacb.com/). Reads were pre-processed using PacBio SMRT Link (version 10.2.0.133434) for adaptor removal and removal of CCS reads with a quality score (Q)<20. This resulted in a total of 1,003,881 reads, summing 12,148,926,671 bases, with 98.4% being >Q20 and 96.4% being >Q30.

### Metagenome assembly and symbiont genome retrieval

The genomes of the lacewing host plus the two bacterial symbionts were assembled as a metagenome using Hifiasm [38] (version 0.19.8-r603), with haplotig duplication purging. This metagenome assembly then underwent three rounds of polishing with the PacBio reads using Racon [39] (version 1.5.0). The resulting draft metagenome, comprising lacewing, *Spiroplasma* and *Rickettsia* genomes*,* consisted of 233 contigs, with a total length of 470 Mb, and an N50 of 71 kb. Contigs were taxonomically assigned to the lacewing, *Spiroplasma* or *Rickettsia* using a combination of Blobtools2 [40] (version 3.0.0), minimap2 [41] (version 2.24-r1122) and blast+ [42] (version 2.12.0+). The *Spiroplasma* and *Rickettsia* metagenome-assembled contigs were separately assessed for genome size, number of contigs, completeness and contamination using QUAST [43] (version 5.0.2), BUSCO [44] (version 5.2.2) and CheckM2 [45] (version 0.1.3). Where duplicated copies of BUSCO genes were inferred, these were manually checked to confirm that two distinct copies were present in the genome (e.g. different sequence and different orientation).

### Genome size comparison

For *Mollicutes*, to which *Spiroplasma* belongs, the size in bp of sequenced complete genomes was collated from the database Molligen4 (https://services.cbib.u-bordeaux.fr/molligen4). For the *Rickettsiales*, to which *Rickettsia* belongs, genome information was extracted from recently published data [46]. The size of a selection of these genomes (107 Mollicutes and 139 Rickettsiales) was plotted using R [47] (version 3.6.3) with the packages ggplot2 [48] (version 3.5.0), dplyr [49] (version 1.1.4) and forcats [50] (version 1.0.0), alongside the symbionts reported here, to visualize the size of focal symbionts in the context of their taxonomic groups.

### Symbiont genome annotation of CDSs and toxin genes

CDSs of the main chromosome for both *Rickettsia* and *Spiroplasma* were identified and annotated using BAKTA [51] (version 1.9.3). The *Spiroplasma* and *Rickettsia* main chromosomes were also functionally annotated using Anvi’o [52] (version 8), running the commands anvi-run-kegg-kofams and then anvi-estimate-metabolism to estimate KEGG pathway completeness. DFAST [53] (version 1.3.0) was employed to ascertain the number of pseudogenes in each genome. Putative plasmid sequences were identified as being circular extra-chromosomal contigs with coverage higher than the main chromosome.

Several genes have been reported to be involved in male-killing, cytoplasmic incompatibility or other symbiont-induced phenotypes such as those relating to a defensive role against parasites, parasitoids or pathogens. We searched the *Spiroplasma* genome for homologues of Spaid, *cif *genes and genes encoding RIP, OTU, ankyrin repeat or ETX/MTX2 domains, while in the *Rickettsia* genome, we searched for *cif* genes. To identify Spaid-like homologues (CDSs that have high sequence similarity to part or all of Spaid) in the genome of *Spiroplasma s*Md, a tBLASTn search was made using the Spaid synthetic construct (accession AWJ64280.1). For genes encoding a RIP, OTU, ankyrin repeat or ETX/MTX2 domain, a combinatorial approach was taken: (1) peptide sequences containing those domains were retrieved from NCBI or provided from the authors [54] and used as queries to search the *Spiroplasma* genome via tBLASTn, and (2) CDS and domain annotation software results were manually checked to identify genes encoding those domains. To ascertain whether either *Spiroplasma s*Md or *Rickettsia r*Md encodes *cif* genes, the genomes of both were searched using tBLASTn using *cifA* and *cifB* protein sequences retrieved from NCBI as queries (*cifA*: WP_010962721.1 and *cifB*: WP_010962722.1).

The blast top hits were then assessed, and domains were predicted using a combination of tools. The presence of signal peptides was predicted using SignalP [55] (https://services.healthtech.dtu.dk/services/SignalP-6.0; version 6.0), and the protein sequences of the top hits searched for domain homology using InterPro [56] (https://www.ebi.ac.uk/interpro/), PHMMER [57] (https://www.ebi.ac.uk/Tools/hmmer/search/phmmer), HHpred [58] (https://toolkit.tuebingen.mpg.de/tools/hhpred), PFAMSCAN [59] (https://www.ebi.ac.uk/jdispatcher/pfa/pfamscan) and SMART [60] (https://smart.embl.de). In some cases, the predicted protein structure was inferred with AlphaFold [61] (https://alphafoldserver.com; version 3) and compared to previously characterized proteins of known function utilizing the AlphaFold Protein Structure Database (https://alphafold.ebi.ac.uk).

### Mobile genetic element and repeat detection

The main chromosomes of the *Spiroplasma* and *Rickettsia* symbionts carried by the lacewing host, along with genomes of congeneric representatives, were analysed for the presence of repeat sequences and common mobile genetic elements. For *Spiroplasma*, 54 additional genomes were included (genome accessions in Table S5, available in the online Supplementary Material), while for *Rickettsia*, 32 additional genomes were included (genome accessions in Table S4). Only genomes where the main chromosome was complete (one contig) were used in the comparative study. The overall repetitive content of the genomes was assessed and visualized using MUMmer4 [62] (version 4.0.0rc1). Prophage regions, insertion sequences (ISs) and tandem repeats were identified using PHASTEST [63] (https://phastest.ca), ISEScan [64] (version 1.7.2.3) and Tandem Repeat Finder [65] (version 4.09.1), respectively. The presence of CRISPR repeats and spacers was investigated using CRISPR-CasFinder [66] with default parameters (https://crisprcas.i2bc.paris-saclay.fr/CrisprCasFinder/Index). The results from these analyses were plotted using R [47] (version 3.6.3) with the packages ggplot2 [48] (version 3.5.0), dplyr [49] (version 1.1.4) and forcats [50] (version 1.0.0). The main chromosomes of *s*Md and *r*Md were visualized using Circos [67] and depict the coordinates of prophage region(s), IS elements, tandem repeats and also the GC content.

For the *Rickettsia* symbiont, the main chromosome was additionally analysed for the presence of ‘Rickettsiales amplified genetic elements’ (RAGEs) as these elements have previously been shown to be important in *Rickettsia* genome expansion events [68,70]. For this, a search was made for the presence of a definitive sequence of *transfer* and associated genes, as described in Giengkam *et al.* [70].

### Phylogenomic analyses

The phylogenetic relationship of the symbionts under study was analysed alongside selected representatives from their respective genera – the same strains as were included in the comparative analysis (Tables S4–S5). The phylogenies for both *Spiroplasma* and *Rickettsia* were based upon sequence data of 67 single-copy core orthologues identified as being present in all genomes, then concatenated and aligned in Anvi’o [52] (version 8). The best protein model was identified using ModelFinder [71], and a maximum likelihood tree was reconstructed in IQTree2 [72] (version 2.3.0) and visualized using FigTree (version 1.4.4, https://github.com/rambaut/figtree). According to the Bayesian Information Criterion, for the *Rickettsia* phylogeny, the best-fit model chosen was Q.plant+F+R4, and for the *Spiroplasma* phylogeny, the best fit model was LG+F+I+R5. *Mycoplasmoides genitalium* and *Orientia tsutsugamushi* were used as outgroups for the *Spiroplasma* and *Rickettsia* trees, respectively.

To ascertain whether particular IS elements were shared between the symbiont genomes, the phylogenetic relationships of transposase sequences from the shared IS families were estimated. For this, where a particular IS family was identified as being found in both bacterial genomes, the longest transposase CDS was chosen from each bacterium, and from selected representatives from the *Spiroplasma* and *Rickettsia* genera. For each IS family, the amino acid sequences were aligned using MEGAX [73] (version 11.0.13). The best protein model was identified using ModelFinder [71], and a maximum likelihood tree was reconstructed in IQTree2 [72] (version 2.3.0) and visualized using FigTree (version 1.4.4, https://github.com/rambaut/figtree).

## Results

### The genomes of *Spiroplasma s*Md and *Rickettsia r*Md

The *Spiroplasma* and *Rickettsia* genomes retrieved from the lacewing metagenome were each assembled into one complete circular bacterial chromosome, plus three circular plasmids for *Rickettsia*. The presence and number of *Spiroplasma* plasmids remain unresolved due to copious repetitive sequences that make their status ambiguous. For *Spiroplasma*, hereafter named strain *s*Md, the main chromosome is 3,137,804 bp, and for *Rickettsia*, hereafter named strain *r*Md, the main chromosome is 3,245,895 bp. CheckM2 and BUSCO analyses indicated that the genomes were 99–100% and 96–100% complete for core marker sets, respectively. The main chromosome of each symbiont genome was annotated to predict the number and type of CDSs (Table 1).

**Table 1.** Symbiont genome main chromosome assembly statistics, completeness and CDS annotation

		*Spiroplasma s*Md	*Rickettsia r*Md
Assembly	Contigs	1	1
	Total length (bp)	3,137,804	3,245,895
	GC (%)	23.51	31.66
	# Ns	0	0
	Circular	Yes	Yes
BUSCO	Complete (%)	99	96
	Single (%)	98	92
	Duplicate (%)	1	4
	Fragment (%)	0	0
	Missing (%)	1	3
	Total orthologues	151	364
	Database	Mollicutes	Rickettsiales
CheckM2	Completeness (%)	99.99	99.51
	Contamination (%)	4.13	6.07
	Completeness model	Neural network	Gradient boost
	Translation table	4	11
Annotation	CDS	4,430	3,818
	rRNA	3	3
	tmRNA	1	1
	tRNA	32	35

### The genomes of *Spiroplasma* and *Rickettsia* contain genes potentially associated with reproductive parasitism and/or protective symbiosis

**Fig. 1.**
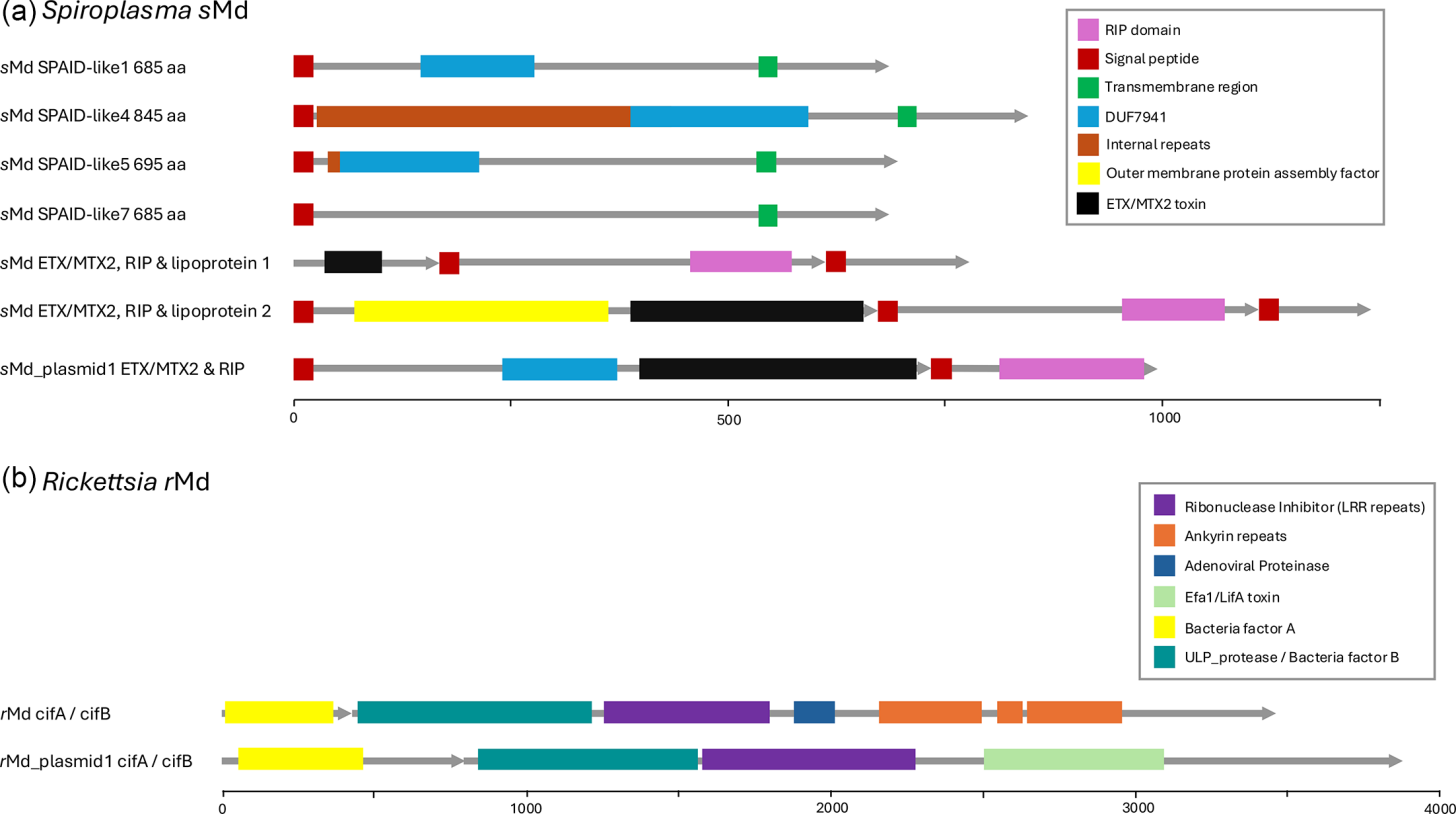
Predicted CDS with putative toxin domains in (a) *Spiroplasma s*Md and (b) *Rickettsia r*Md. The x-axis represents amino acid residues.

In the *Spiroplasma s*Md genome, four ORFs were identified by blast homology as having similarity to Spaid primarily at the C-terminal hydrophobic region (SPAID-like1: score=285, e-value=5e-79, ID=73%; SPAID-like4: score=276, e-value=3e-76, ID=63%; SPAID-like5: score=273, e-value=2e-75, ID=72%; SPAID-like7: score=261, e-value=1e-71, ID=72%). The predicted proteins each had signal peptide sequences and C-terminal transmembrane domains (Fig. 1a). However, they lacked ankyrin repeats and OTU domains that are necessary for Spaid function as a male-killer in *D. melanogaster* [25]. Three of the four Spaid-like proteins carry the same Domain of Unknown Function (DUF7941). A search on InterPro revealed that this domain is widely present in *Uroviricota* tailed phage and *Salmonella* and also found in three apis group *Spiroplasma* species isolated from insect guts (*S. litorale*, *S. helicoides* and *S. corruscae*). However, this domain is novel to heritable *Spiroplasma* strains and has no known virulence association.

Two other islands of interest were noted on the main chromosome which each composed a trio of genes: one encoding an ETX/MTX2 toxin domain, a second with a RIP domain and a lipoprotein (Fig. 1a). In one island, the three predicted proteins all have signal peptides. In the second island, the predicted ETX/MTX2 domain protein lacks a signal peptide. Examination of the putative *s*Md plasmid revealed a similar unit of one predicted ETX/MTX2 domain protein alongside a predicted RIP protein (both with a signal peptide), potentially in two tandemly arranged copies, but without the downstream lipoprotein-coding gene (Fig. 1a). AlphaFold predicted that the ETX/MTX2 domain proteins were all non-globular and resembled canonical ETX/MTX2 from *Clostridium perfringens* in terms of an elongated beta-pleated sheet element (Fig. S1).

In the *Rickettsia r*Md genome, we identified one *cifA/cifB* pair on the main chromosome (Fig. 1b). The predicted CifA protein is 427 amino acids, whereas CifB is 3,036 amino acids. In addition, one of the plasmids of *r*Md contains a *cifA/cifB* dyad, distinct from the chromosomal version in sequence and some of the domains present (Fig. 1b). The *cifA* and *cifB* homologues identified in the main chromosome and plasmid were also predicted to be similar in structure to regions of the complex formed by *Wolbachia w*Pip cytoplasmic incompatibility factors *CinA* and *CinB* [74] [namely 7ET0 and 7FIW in the RCSB Protein Data Bank (https://www.rcsb.org)]. No *cif* gene homologues were found in *Spiroplasma s*Md.

### The *Spiroplasma* and *Rickettsia* genomes are the largest within their respective clades due to proliferation of repetitive elements

Both of the symbiont genomes are, to our knowledge, the largest complete genomes sequenced to date in their respective genera. Moreover, *Spiroplasma s*Md is the largest sequenced closed genome within the class Mollicutes (Fig. S2), and *Rickettsia r*Md is the largest sequenced complete genome within the order Rickettsiales (Fig. S3).

**Fig. 2.**
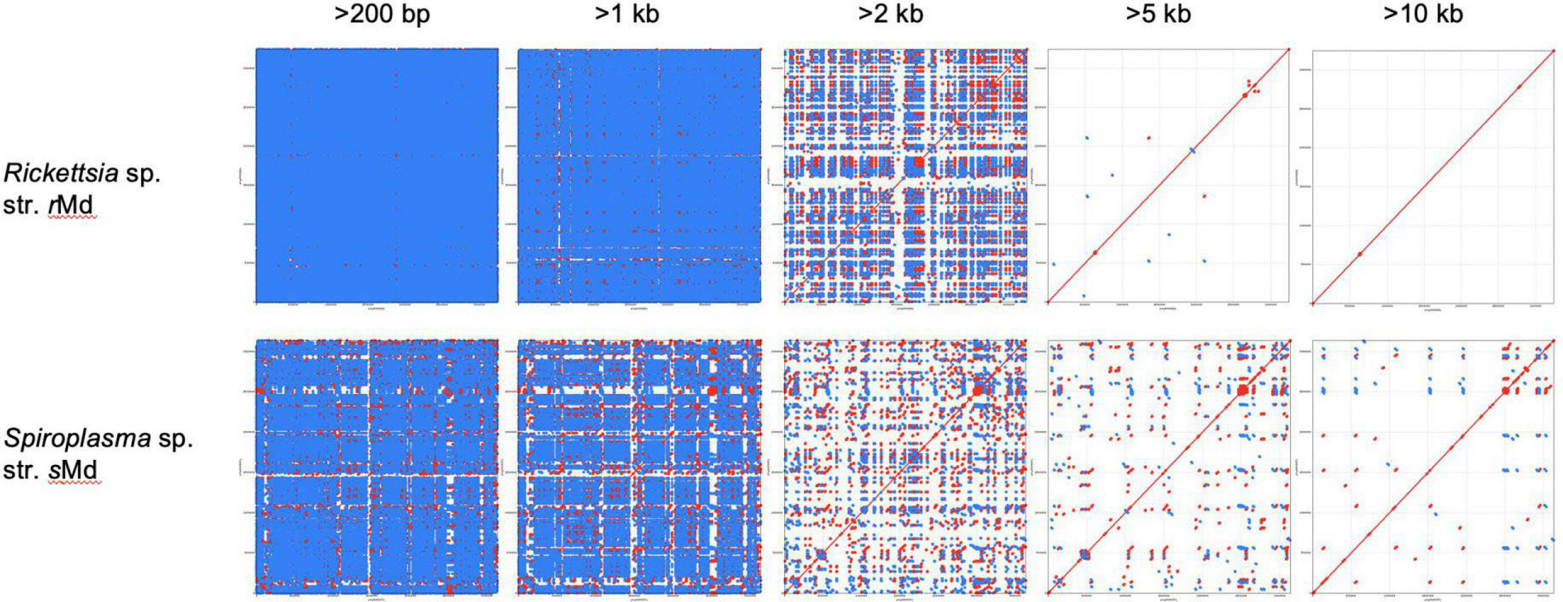
MUMmer plots depicting repeat sequences within the *Spiroplasma s*Md and *Rickettsia r*Md main chromosomes. Repeats were filtered by size and then plotted. For each graph, the x-axis and the y-axis are the same; i.e. the main chromosome is plotted against itself. Repeats are shown in blue (forward direction) and red (reverse direction).

In order to investigate the factors driving the size expansion of both of the symbiont main chromosomes, we analysed whether the genomes contained repeat sequences. The BUSCO results indicate that core genes of *Spiroplasma* are rarely duplicated (1 out of 151 genes) with some evidence of core gene duplication in *Rickettsia* (15 out of 364 genes, verified manually) (Table 1). The main chromosomes of both symbionts show very high levels of repetition (Fig. 2). Observation of the plots reveals a difference in the repeat pattern between the main chromosomes of *Spiroplasma* and *Rickettsia. Rickettsia* is dominated by repeats 200–5,000 bp in length, while *Spiroplasma* additionally has a larger number of repeats in the >5 kb range.

To ascertain the level of diversity in repetitiveness across *Spiroplasma* and *Rickettsia*, repetitive sequences in the main chromosome of four representatives from each genus were similarly visualized using mummerplot and compared to the strains isolated from the lacewing (Figs S4 and S5). Our focal symbionts displayed a broader signal of repetition than other members of the genus.

### The *Spiroplasma* and *Rickettsia* genomes are dominated by ISs and/or prophage

**Fig. 3.**
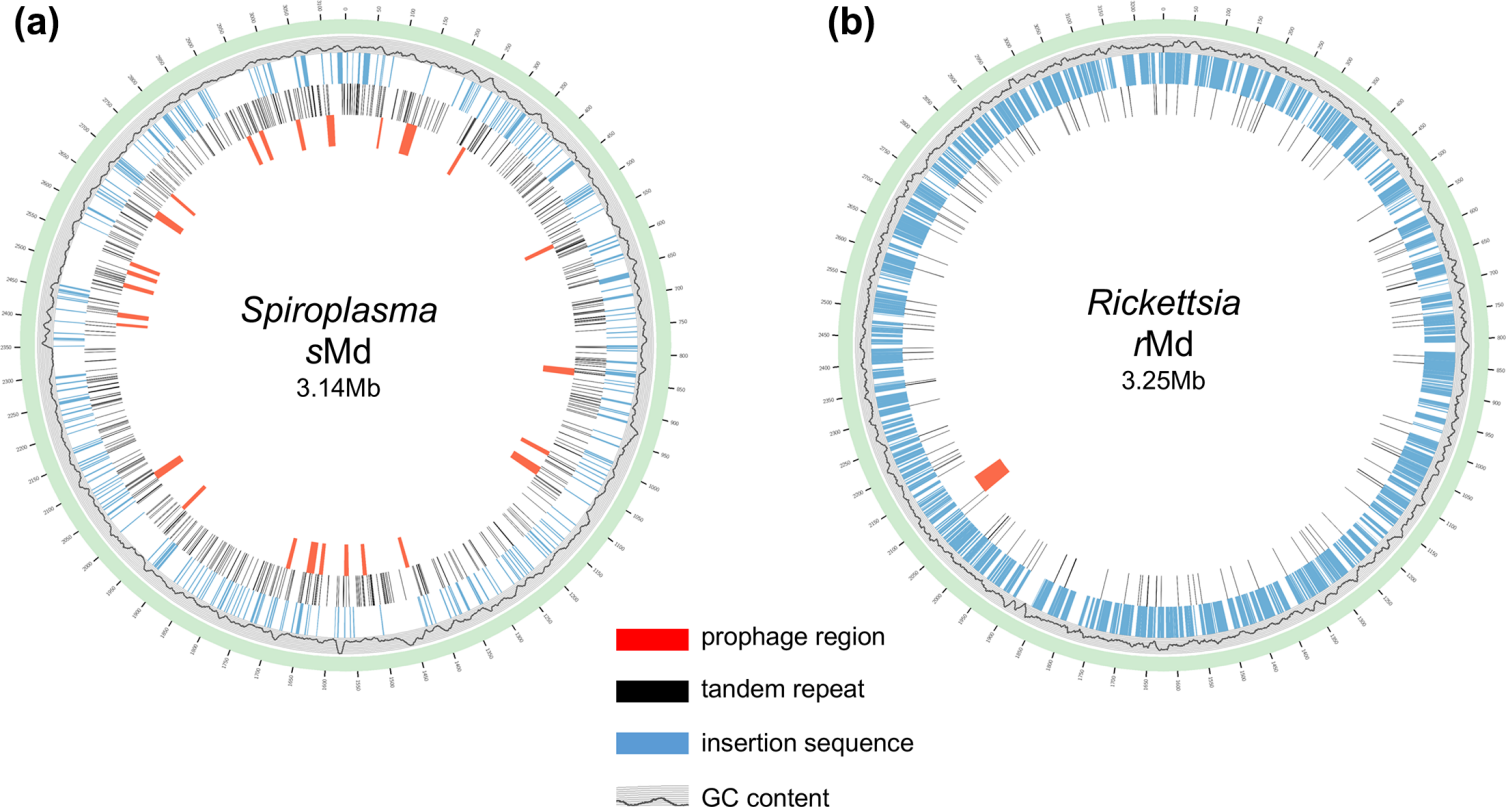
Circos plots depicting GC content and mobile genetic element features of the (**a)** *Spiroplasma s*Md and (**b)** *Rickettsia r*Md main chromosomes.

The *Spiroplasma s*Md main chromosome has 4,430 CDSs, with 407 being transposases (just under 10% of the CDS; Table S1). There were 309 full ISs, comprising transposase plus flanking repeats, totalling 432,487 bp. In addition, 1,177 of the CDSs annotated were assigned as being phage-related (contain keywords: phage, virus, capsid, integrase, terminase, baseplate), making up ~25% of the CDS (Table S1). When the presence of prophage within the *Spiroplasma* main chromosome was investigated further, 26 phage regions were inferred, totalling 276.1 kb. Further repetitive sequences were identified in the form of 621 tandem repeats (Fig. 3a). Again, there was no evidence of a CRISPR-Cas system.

Annotation of the *Rickettsia r*Md main chromosome revealed that of 3,818 CDSs, almost one third of these (1,114) were classed as transposases or IS accessory proteins (Table S2). When analysing the genome for full IS, which typically comprises a transposase flanked by short repeat sequences, the *Rickettsia* main chromosome carried 1,564 ISs totalling 1,778,924 bp and comprised 54.81% of the main chromosome. In addition to IS, *Rickettsia* also had 142 tandem repeats and 1 intact phage region of 41.4 kb (Fig. 3b). While a single region of multiple *transfer* genes in the *Rickettsia* genome indicates the presence of a putative RAGE, it is unclear whether this is functional given interruption by numerous transposases. There was no evidence of a CRISPR-Cas system.

### *Spiroplasma* and *Rickettsia* do not share IS

Given the presence of large numbers of ISs in both the *Spiroplasma* and *Rickettsia* main chromosomes, we investigated whether the particular ISs found were shared between the two bacterial strains, reflecting lateral gene transfer as a driving factor of the concerted genome expansions. The ISs situated in the main chromosome of *Rickettsia* belong to 17 different IS families (Table S3). Four of these IS families are shared with those found in *Spiroplasma* (IS3, IS5, IS30 and IS481) – with these four being the only IS families present in *Spiroplasma*. However, the frequencies of the IS differ between the two symbionts. For *Spiroplasma*, by far the most prevalent ISs are from family IS30 (*n*=269), fewer from IS481 (*n*=33), and IS3 and IS5 are rare (*n*=3 and *n*=4, respectively). In contrast, of these four IS families, the reverse is true; in *Rickettsia*, IS3 is the most prevalent (*n*=506), then IS5 (*n*=23) and then IS481 (*n*=21), with IS30 being the least prevalent (*n*=7).

For both *Spiroplasma* and *Rickettsia*, transposase CDSs from each of the four IS families were aligned alongside sequences from selected congeneric strains, and four phylogenies were estimated. For each IS family, the transposase sequences cluster with those from their respective bacterial genera rather than with each other (Figs S6–S9). Taken together, the results indicate that the IS elements are not shared by *Spiroplasma* and *Rickettsia* co-infecting the lacewing host by direct transfer from one symbiont to the other.

### *Spiroplasma* and *Rickettsia* genomes retain a high level of functionality

There was a high level of pseudogenization in *Rickettsia r*Md and a moderate level in *Spiroplasma s*Md as ascertained by DFAST (*Rickettsia* 1,179 pseudogenes, 926 of which are transposases; *Spiroplasma* 286 pseudogenes, 40 of which are transposases). However, KEGG analysis reveals that both genomes retain a high level of functionality, with module completeness being similar to other members of their respective genera (Figs S10–14).

### IS elements and prophage regions vary in presence and frequency across *Spiroplasma* and *Rickettsia*

**Fig. 4.**
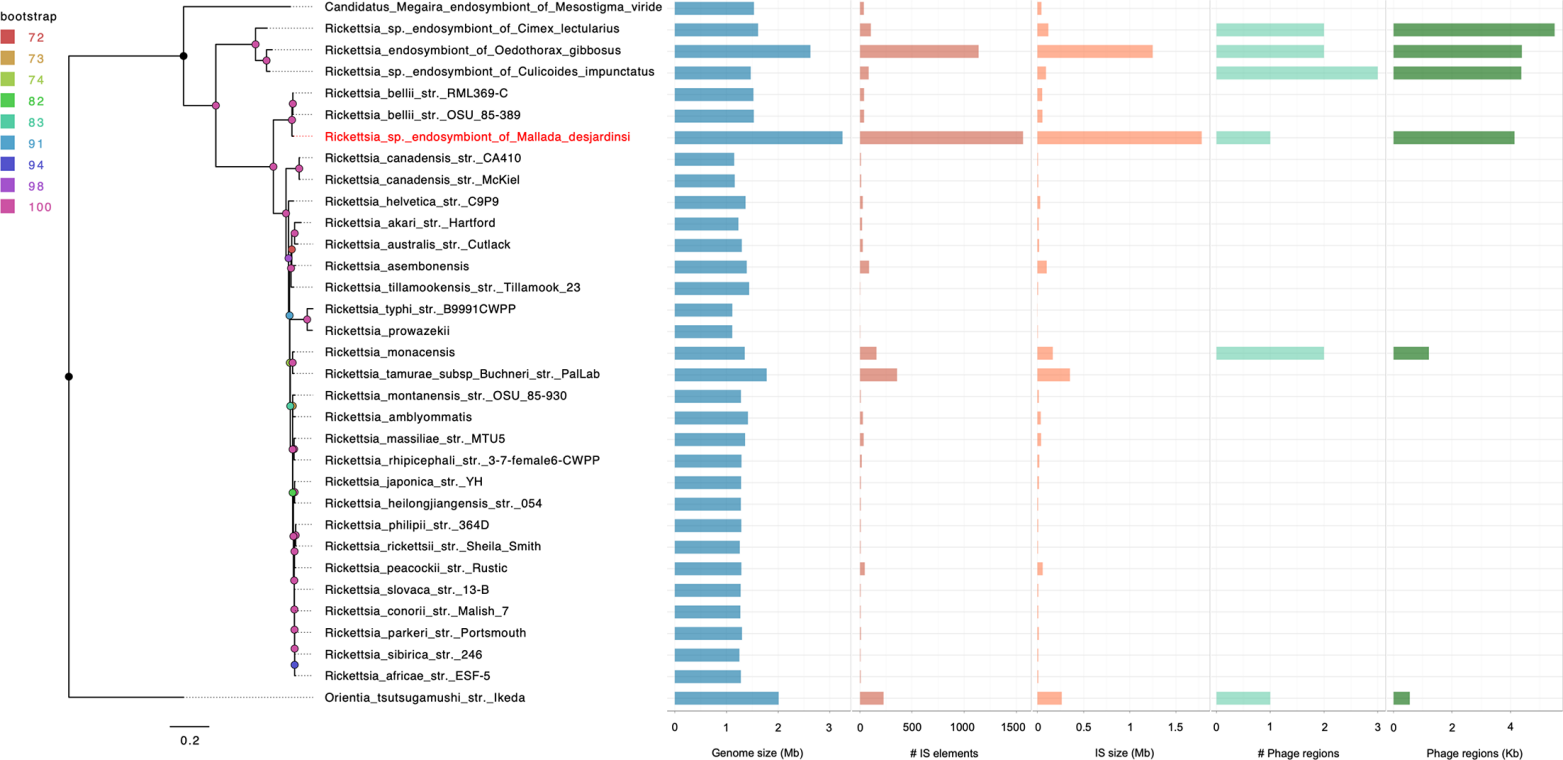
Phylogeny of *r*Md (red) and selected *Rickettsia* strains with sequenced complete genomes. Genome size (Mb), the number of IS elements along with the total size of IS (Mb) and the number of phage regions with total size (kb) are also given alongside.

To investigate whether there is a general relationship between main chromosome size and the presence of IS and prophage, a selection of sequenced complete genomes (main chromosome in one contig) was analysed and compared to that of the *Rickettsia* strain *r*Md isolated from the lacewing *M. desjardinsi* (Fig. 4, Table S4). *Rickettsia r*Md lies within the bellii group of *Rickettsia*, which comprises both vertically inherited and pathogenic bacteria. As previously mentioned, this *Rickettsia* has the largest genome in the genus, and also the highest number of IS elements. Tellingly, the second largest *Rickettsia* strain, isolated from the spider *Oedothorax gibbosus* at 2.62 Mb [75], has the second largest number of IS, indicating that proliferation of IS also drove genome expansion in this bacterium. While the lacewing *Rickettsia* does have one prophage, it is not a driving factor in its genome expansion, and other smaller genomes within *Rickettsia* have higher numbers of prophage regions.

**Fig. 5.**
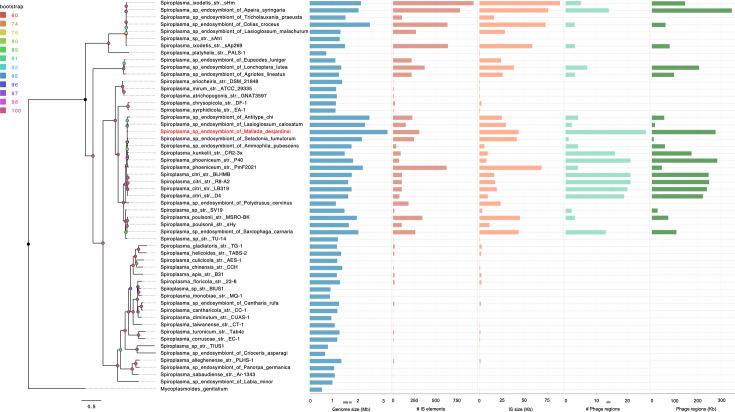
Phylogeny of *s*Md (red) and selected *Spiroplasma* strains with sequenced complete genomes. Genome size (Mb), the number of IS elements along with the total size of IS (Mb) and the number of phage regions with total size (kb) are also given alongside.

Similarly, a selection of sequenced complete *Spiroplasma* genomes (main chromosome in one contig) were analysed and compared to that of the *Spiroplasma* strain *s*Md isolated from the lacewing *M. desjardinsi* (Fig. 5; Table S5). *Spiroplasma s*Md is most closely related to infectiously transmitted insect-vectored plant pathogens, including *S. citri* and *S. phoeniceum* [18]. As previously stated, *s*Md has the largest main chromosome sequenced as a complete genome to date. While it does have numerous IS, others within the genus have comparable or higher numbers of IS. While the proliferation of IS may be a contributing factor driving genome expansion in *s*Md, it is a contributor alongside other repetitive elements. *Spiroplasma s*Md does, however, have the highest number of prophage regions, although the total size is not the highest observed in the genus. Looking across the *Spiroplasma* strains, it appears that a combination of proliferation of IS and prophage leads to genome expansion within this genus.

## Discussion

Our research was motivated by the potential insight genome sequences allow into the evolution of symbionts, and to develop hypotheses for the mechanisms of phenotypes. Our *Spiroplasma* male-killer genome carried multiple copies of genes whose predicted proteins have sequence similarity to the C-terminal of Spaid, the male-killing toxin of *Spiroplasma poulsonii* in *D. melanogaster*. However, the sequence similarity of these proteins to Spaid did not extend to the N-terminal ankyrin and OTU domains known to be necessary for male-killing [25]. Thus, the causal basis of male-killing by *s*Md differs from that of *s*Mel in *D. melanogaster*. This diversity of male-killing effectors likely reflects differences in sex determination between the species and reflects the diversity of genes underpinning the phenotype across symbionts and symbioses. The actual male-killing factor in *s*Md is uncertain.

One of the surprising features of the sMd genome was the contrast between a powerful phenotype (male-killing) and the lack of diverse effector systems in the genome. Because predicted effectors were rare, those that were present warrant onward investigation. Most notable of these is a dyad of toxin genes present twice on the main chromosome, and also on a putative plasmid. In each case, a gene encoding an RIP domain was preceded by one encoding an ETX/MTX2 domain, and in two cases on the main chromosome, these were followed by a lipoprotein. Previous work indicates the RIP domain as a candidate for interference at the eukaryotic ribosome [30], and ETX/MTX2 domain proteins as forming a heptamer that creates a beta barrel pore in the eukaryotic plasma membrane [76]. The predicted protein structure of *s*Md ETX/MTX2 domain proteins had a strong resemblance to the structure of the canonical ETX/MTX2. There is no obvious link of these gene functions to male-killing, but the repetition of this array of effectors bears onward functional investigation.

Genomic analysis indicates that the *Rickettsia* – whose phenotype is currently uncharacterized – contains a pair of *cifA/cifB-*like genes, one on the main chromosome and one on a plasmid. This gene dyad is commonly associated with cytoplasmic incompatibility, which has recently been reported as a phenotype of *Rickettsia* in insects [36]. However, it should be noted that the capacity of a symbiont to induce CI can be host dependent – thus, the actual presence of CI remains to be determined through crossing studies.

While not the initial intention of our study, the *Spiroplasma* and *Rickettsia* genomes from the lacewing were notable for their particularly large size. At 3.25 Mb, the main *Rickettsia* genome is the largest recorded to date in the Rickettsiales and also harbours the largest plasmids in the family, two of which (448 and 172 kb) exceed the size of the previously recorded largest plasmid in *Ixodes pacificus* at 121 kb [77]. At 3.14 Mb, the *Spiroplasma* is also significantly enlarged, with the largest closed circle reported to date (though we note the fragmentary assembly of *Spiroplasma* from *Morpho* butterflies, which is comparable [78]). For both clades, typical genome sizes are 1–2 Mb.

The evolution of symbiont genomes is generally considered reductive. Genome streamlining is driven by the reduced effective population size of symbionts reducing the efficacy of purifying selection compared to free-living relatives, combined with a bias to deletion mutations in microbial genomes. These processes result in progressive pseudogenization and ultimately smaller genomes of very high coding density [79,80]. Secondary genome expansion – where streamlined genomes increase in size – was first observed in a member of the Rickettsiales, *O. tsutsugamushi*, whose 2.0–2.5 Mb genome was considerably larger than other members of the bacterial order (typically 1–1.5 Mb) [68,81]. In *Orientia*, the accumulation of mobile genetic elements and accessory genes such as transposases, *transfer* genes, phage integrases and reverse transcriptases and proliferation of an integrative and conjugative element called ‘Rickettsiales amplified genetic element’ (RAGE) led to repetitive elements comprising almost 50% of the genome [68,70]. A further study revealed the particularly large genome of another rickettsial symbiont with its ~1.8 Mb genome comprising ~35% RAGEs alongside transposases and other MGEs [69]. More modest genome expansions have also been observed for other symbionts such as *Wolbachia* and *Amoebophilus asiaticus* [82,84].

The proximate causes of genome expansion in our co-infecting microbes were distinct – *Spiroplasma* genome expansion was largely driven by prophage accumulation, *Rickettsia* by diverse IS elements. We can thus exclude a common exposure to horizontally transmitted elements – the intracellular arena scenario [85] – as a driver of our patterns of concerted genome expansion. The secondary genome expansion events thus represent a convergent phenotype associated with diverse repetitive elements. Moreover, the IS-mediated genome expansion of *Rickettsia r*Md is distinct from the genome expansion of *Orientia* and other *Rickettsia*, in being associated with a single mobile element type (rather than combinations of IS elements and RAGE). Genome expansion of *Spiroplasma sMd* resembles that of the *Spiroplasma* from *Morpho* butterflies, which also included both IS and prophage proliferation [78]. It is also notable that the *s*Md is closely related to the strains present in *Morpho*, potentially indicating that these genomes are particularly prone to mobile element expansions.

Our observation of concerted genome expansion, two symbionts within a host species independently increasing in genome size, is notable. However, its significance is unclear. Currently, we do not understand why secondary genome expansions are observed in particular lineages. A null hypothesis is that expansions are random with respect to the lineages where expansion occurs – perhaps consequent on a stochastic lateral transfer of a mobile element that can then proliferate. The alternate hypothesis is that there are particular circumstances that enable proliferation. For instance, both IS movement and phage activation are induced by stress on the host microbe. One prediction of the alternate hypothesis of host-driven factors is that heritable symbionts that co-infect a host would be predicted to have correlated evolutionary trajectories. Put simply, if the host provides the context driving genome expansion, it may be seen in both co-infecting symbionts. Our concerted genome expansion events are thus consistent with an underpinning driver.

One observation from our work is that heritable microbes can tolerate these high loads of mobile elements, which, in the case of the *Rickettsia*, comprise around half the genome and a doubling in size compared to most members of the genus. KEGG analysis indicated no obvious degradation of symbiont metabolic function compared to non-expanded relatives. Thus, purifying selection remained sufficiently strong to maintain the function of core genes. Heritable microbes experience both within- and between-host selection [86], and it is expected that mutations causing loss of core functions would compromise within-host competitiveness and thus be eliminated. The *Rickettsia* data also suggest a potential runaway scenario, where the build-up of mobile genetic content makes onward IS insertions more likely to occur outside of bacterial core genes, and thus less likely to be disruptive. In this model, expansion creates positive feedback by creating non-functional islands into which further insertions can occur without deleterious effects. For the *Spiroplasma*, the novel prophage material may have both costs and benefits. On the one hand, prophage adds genetic material and may become lytic if the host is stressed. Contrastingly, they commonly carry a cargo of symbiosis-relevant genes, as observed in the *Arsenophonus* genome expansion [87].

In conclusion, our symbiont genome sequencing indicates that male-killing by *Spiroplasma* in *M. desjardinsi* does not involve Spaid, in the manner found in the *S. poulsonii*/*D. melanogaster* symbiosis. The *Rickettsia* genome sequence indicates that it may cause cytoplasmic incompatibility. Most markedly, we observed a case of concerted secondary genome expansion. Our observation of high levels of expansion in two co-infecting symbionts, with both recorded as the largest in their bacterial groups, is suggestive of a core underpinning cause of secondary genome expansion events.

## Supplementary material

10.1099/mgen.0.001766 Supplementary Material 1.

10.1099/mgen.0.001766 Supplementary Material 2.

## Abbreviations

CI: cytoplasmic incompatibility
ISs: insertion sequences
RAGEs: Rickettsiales amplified genetic elements
RIP: ribosome-inactivating protein
