# Supplementary material for: Analysis of the genomes of a male-killing Spiroplasma and its co-infecting Rickettsia reveals a case of concerted genome expansion
Source: Microb Genom. 2026 Jul 16;12(7):001766. doi: 10.1099/mgen.0.001766 (PMC13375240; doi:10.1099/mgen.0.001766)
Supplement: Supplementary Material 1. [file mgen-12-01766-s001.pdf]

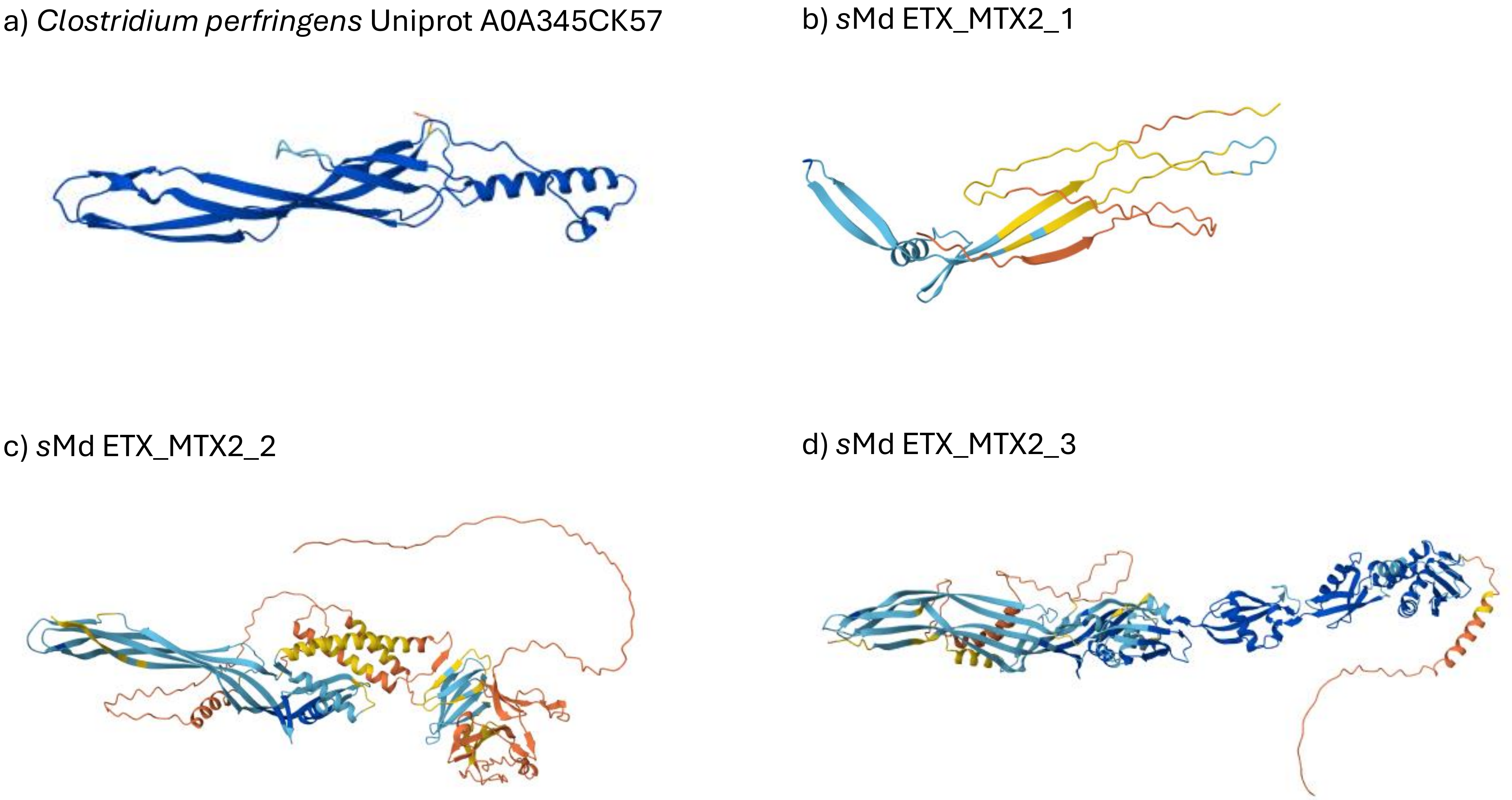


**Figure S1.** AlphaFold predicted structures for a) Canonical ETX/MTX2 toxin from C. perfringens and b)-d) putative ETX/MTX2 toxins from sMd. Colours indicate certainty of structure (dark – certain, light – uncertain)

**Figure S2**

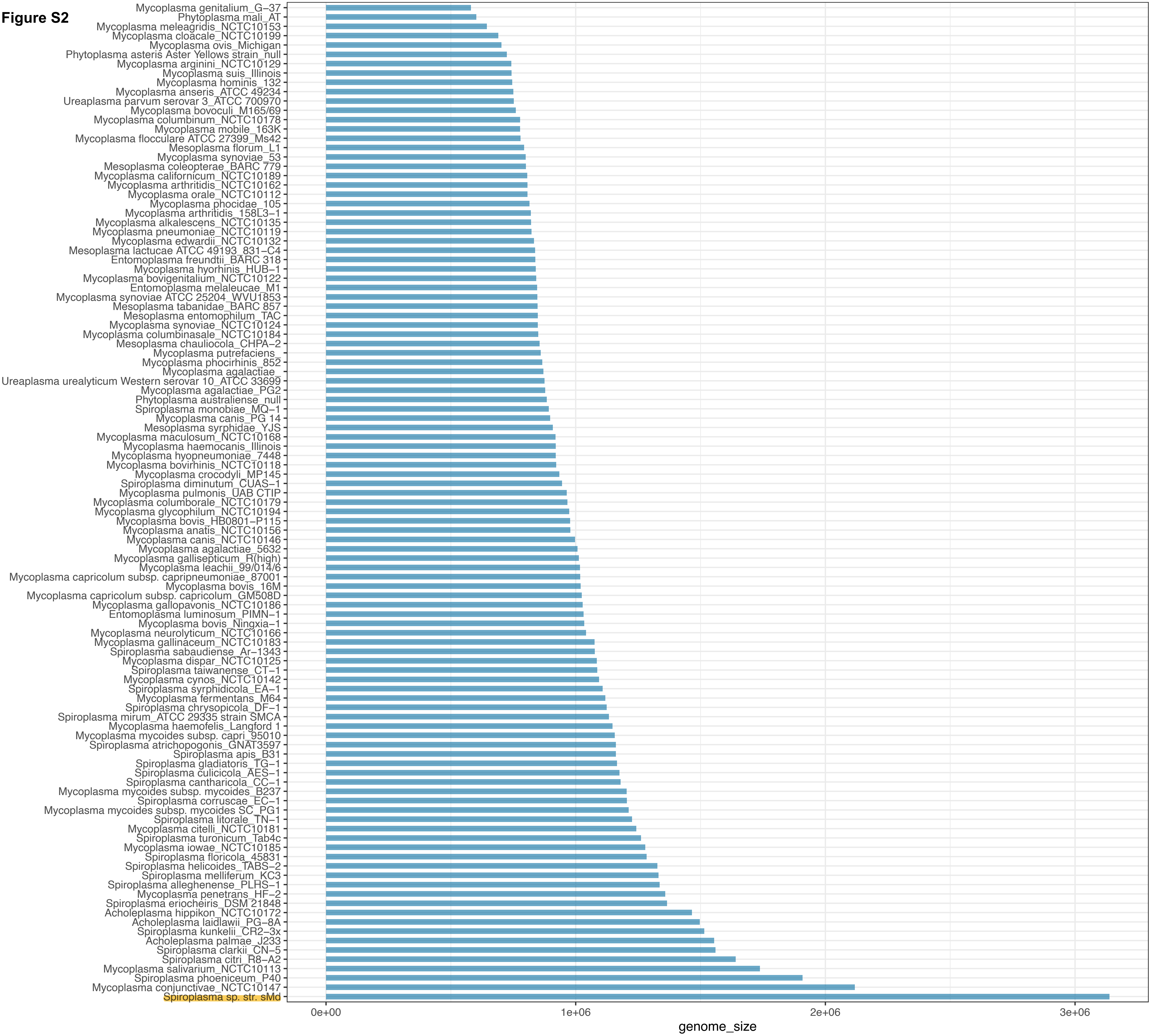
Mycoplasma genitalium_G−37
Phytoplasma mali_AT
Mycoplasma meleagridis_NCTC10153
Mycoplasma cloacale_NCTC10199
Mycoplasma ovis_Michigan
Phytoplasma asteris Aster Yellows strain_null
Mycoplasma arginini_NCTC10129
Mycoplasma suis_Illinois
Mycoplasma hominis_132
Mycoplasma anseris_ATCC 49234
Ureaplasma parvum serovar 3_ATCC 700970
Mycoplasma bovoculi_M165/69
Mycoplasma columbinum_NCTC10178
Mycoplasma mobile_163K
Mycoplasma flocculare ATCC 27399_Ms42
Mesoplasma florum_L1
Mycoplasma synoviae_53
Mesoplasma coleopterae_BARC 779
Mycoplasma californicum_NCTC10189
Mycoplasma arthritidis_NCTC10162
Mycoplasma orale_NCTC10112
Mycoplasma phocidae_105
Mycoplasma arthritidis_158L3−1
Mycoplasma alkalescens_NCTC10135
Mycoplasma pneumoniae_NCTC10119
Mycoplasma edwardii_NCTC10132
Mesoplasma lactucae ATCC 49193_831−C4
Entomoplasma freundtii_BARC 318
Mycoplasma hyorhinis_HUB−1
Mycoplasma bovigenitalium_NCTC10122
Entomoplasma melaleucae_M1
Mycoplasma synoviae ATCC 25204_WVU1853
Mesoplasma tabanidae_BARC 857
Mesoplasma entomophilum_TAC
Mycoplasma synoviae_NCTC10124
Mycoplasma columbinasale_NCTC10184
Mesoplasma chauliocola_CHPA−2
Mycoplasma putrefaciens_
Mycoplasma phocirhinis_852
Mycoplasma agalactiae_
Ureaplasma urealyticum Western serovar 10_ATCC 33699
Mycoplasma agalactiae_PG2
Phytoplasma australiense_null
Spiroplasma monobiae_MQ−1
Mycoplasma canis_PG 14
Mesoplasma syrphidae_YJS
Mycoplasma maculosum_NCTC10168
Mycoplasma haemocanis_Illinois
Mycoplasma hyopneumoniae_7448
Mycoplasma bovirhinis_NCTC10118
Mycoplasma crocodyli_MP145
Spiroplasma diminutum_CUAS−1
Mycoplasma pulmonis_UAB CTIP
Mycoplasma columborale_NCTC10179
Mycoplasma glycophilum_NCTC10194
Mycoplasma bovis_HB0801−P115
Mycoplasma anatis_NCTC10156
Mycoplasma canis_NCTC10146
Mycoplasma agalactiae_5632
Mycoplasma gallisepticum_R(high)
Mycoplasma leachii_99/014/6
Mycoplasma capricolum subsp. capripneumoniae_87001
Mycoplasma bovis_16M
Mycoplasma capricolum subsp. capricolum_GM508D
Mycoplasma gallopavonis_NCTC10186
Entomoplasma luminosum_PIMN−1
Mycoplasma bovis_Ningxia−1
Mycoplasma neurolyticum_NCTC10166
Mycoplasma gallinaceum_NCTC10183
Spiroplasma sabaudiense_Ar−1343
Mycoplasma dispar_NCTC10125
Spiroplasma taiwanense_CT−1
Mycoplasma cynos_NCTC10142
Spiroplasma syrphidicola_EA−1
Mycoplasma fermentans_M64
Spiroplasma chrysopicola_DF−1
Spiroplasma mirum_ATCC 29335 strain SMCA
Mycoplasma haemofelis_Langford 1
Mycoplasma mycoides subsp. capri_95010
Spiroplasma atrichopogonis_GNAT3597
Spiroplasma apis_B31
Spiroplasma gladiatoris_TG−1
Spiroplasma culicicola_AES−1
Spiroplasma cantharicola_CC−1
Mycoplasma mycoides subsp. mycoides_B237
Spiroplasma corruscae_EC−1
Mycoplasma mycoides subsp. mycoides SC_PG1
Spiroplasma litorale_TN−1
Mycoplasma citelli_NCTC10181
Spiroplasma turonicum_Tab4c
Mycoplasma iowae_NCTC10185
Spiroplasma floricola_45831
Spiroplasma helicoides_TABS−2
Spiroplasma melliferum_KC3
Spiroplasma alleghenense_PLHS−1
Mycoplasma penetrans_HF−2
Spiroplasma eriocheiris_DSM 21848
Acholeplasma hippikon_NCTC10172
Acholeplasma laidlawii_PG−8A
Spiroplasma kunkelii_CR2−3x
Acholeplasma palmae_J233
Spiroplasma clarkii_CN−5
Spiroplasma citri_R8−A2
Mycoplasma salivarium_NCTC10113
Spiroplasma phoeniceum_P40
Mycoplasma conjunctivae_NCTC10147
Spiroplasma sp. str. sMd
0e+00
1e+06
2e+06
3e+06
genome_size

**Figure S3**

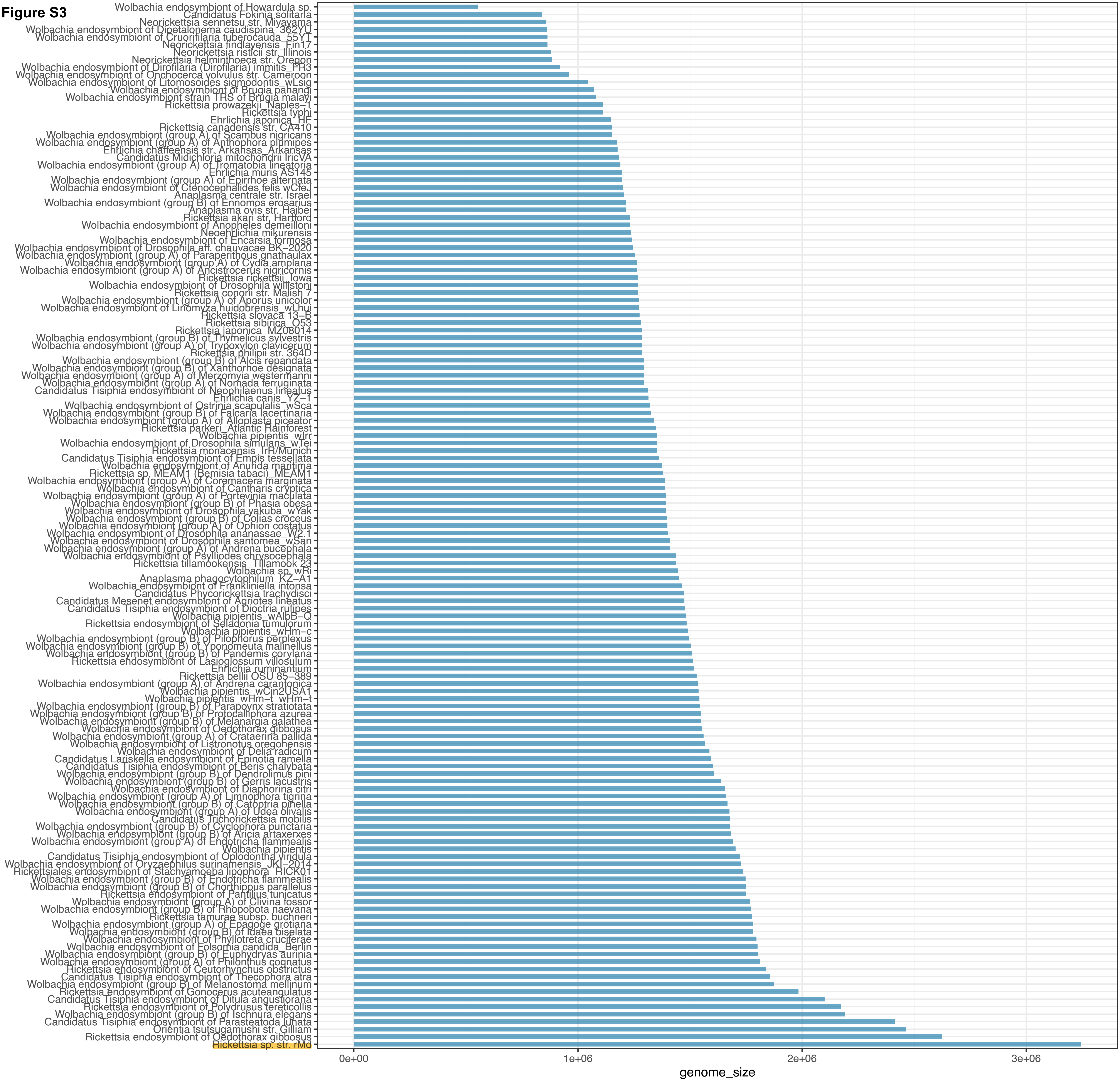

Wolbachia endosymbiont of Howardula sp.
Candidatus Fokinia solitaria
Neorickettsia sennetsu str. Miyayama
Wolbachia endosymbiont of Dipetalonema caudispina_362YU
Wolbachia endosymbiont of Cruorifilaria tuberocauda_55YT
Neorickettsia findlayensis_Fin17
Neorickettsia risticii str. Illinois
Neorickettsia helminthoeca str. Oregon
Wolbachia endosymbiont of Dirofilaria (Dirofilaria) immitis_FR3
Wolbachia endosymbiont of Onchocerca volvulus str. Cameroon
Wolbachia endosymbiont of Litomosoides sigmodontis_wLsig
Wolbachia endosymbiont of Brugia pahangi
Wolbachia endosymbiont strain TRS of Brugia malayi
Rickettsia prowazekii_Naples−1
Rickettsia typhi
Ehrlichia japonica_HF
Rickettsia canadensis str. CA410
Wolbachia endosymbiont (group A) of Scambus nigricans
Wolbachia endosymbiont (group A) of Anthophora plumipes
Ehrlichia chaffeensis str. Arkansas_Arkansas
Candidatus Midichloria mitochondrii IricVA
Wolbachia endosymbiont (group A) of Tromatobia lineatoria
Ehrlichia muris AS145
Wolbachia endosymbiont (group A) of Epirrhoe alternata
Wolbachia endosymbiont of Ctenocephalides felis wCfeJ
Anaplasma centrale str. Israel
Wolbachia endosymbiont (group B) of Ennomos erosarius
Anaplasma ovis str. Haibei
Rickettsia akari str. Hartford
Wolbachia endosymbiont of Anopheles demeilloni
Neoehrlichia mikurensis
Wolbachia endosymbiont of Encarsia formosa
Wolbachia endosymbiont of Drosophila aff. chauvacae BK−2020
Wolbachia endosymbiont (group A) of Paraperithous gnathaulax
Wolbachia endosymbiont (group A) of Cydia amplana
Wolbachia endosymbiont (group A) of Ancistrocerus nigricornis
Rickettsia rickettsii_Iowa
Wolbachia endosymbiont of Drosophila willistoni
Rickettsia conorii str. Malish 7
Wolbachia endosymbiont (group A) of Aporus unicolor
Wolbachia endosymbiont of Liriomyza huidobrensis_wLhui
Rickettsia slovaca 13−B
Rickettsia sibirica_053
Rickettsia japonica_MZ08014
Wolbachia endosymbiont (group B) of Thymelicus sylvestris
Wolbachia endosymbiont (group A) of Trypoxylon clavicerum
Rickettsia philipii str. 364D
Wolbachia endosymbiont (group B) of Alcis repandata
Wolbachia endosymbiont (group B) of Xanthorhoe designata
Wolbachia endosymbiont (group A) of Merzomyia westermanni
Wolbachia endosymbiont (group A) of Nomada ferruginata
Candidatus Tisiphia endosymbiont of Neophilaenus lineatus
Ehrlichia canis_YZ−1
Wolbachia endosymbiont of Ostrinia scapulalis_wSca
Wolbachia endosymbiont (group B) of Falcaria lacertinaria
Wolbachia endosymbiont (group A) of Alloplasta piceator
Rickettsia parkeri_Atlantic Rainforest
Wolbachia pipientis_wIrr
Wolbachia endosymbiont of Drosophila simulans_wTei
Rickettsia monacensis_IrR/Munich
Candidatus Tisiphia endosymbiont of Empis tessellata
Wolbachia endosymbiont of Anurida maritima
Rickettsia sp. MEAM1 (Bemisia tabaci)_MEAM1
Wolbachia endosymbiont (group A) of Coremacera marginata
Wolbachia endosymbiont of Cantharis cryptica
Wolbachia endosymbiont (group A) of Portevinia maculata
Wolbachia endosymbiont (group B) of Phasia obesa
Wolbachia endosymbiont of Drosophila yakuba_wYak
Wolbachia endosymbiont (group B) of Colias croceus
Wolbachia endosymbiont (group A) of Ophion costatus
Wolbachia endosymbiont of Drosophila ananassae_W2.1
Wolbachia endosymbiont of Drosophila santomea_wSan
Wolbachia endosymbiont (group A) of Andrena bucephala
Wolbachia endosymbiont of Psylliodes chrysocephala
Rickettsia tillamookensis_Tillamook 23
Wolbachia sp. wRi
Anaplasma phagocytophilum_KZ−A1
Wolbachia endosymbiont of Frankliniella intonsa
Candidatus Phycorickettsia trachydisci
Candidatus Mesenet endosymbiont of Agriotes lineatus
Candidatus Tisiphia endosymbiont of Dioctria rufipes
Wolbachia pipientis_wAlbB−Q
Rickettsia endosymbiont of Seladonia tumulorum
Wolbachia pipientis_wHm−c
Wolbachia endosymbiont (group B) of Pilophorus perplexus
Wolbachia endosymbiont (group B) of Yponomeuta malinellus
Wolbachia endosymbiont (group B) of Pandemis corylana
Rickettsia endosymbiont of Lasioglossum villosulum
Ehrlichia ruminantium
Rickettsia bellii OSU 85−389
Wolbachia endosymbiont (group A) of Andrena carantonica
Wolbachia pipientis_wCin2USA1
Wolbachia pipientis_wHm−t_wHm−t
Wolbachia endosymbiont (group B) of Parapoynx stratiotata
Wolbachia endosymbiont (group B) of Protocalliphora azurea
Wolbachia endosymbiont (group B) of Melanargia galathea
Wolbachia endosymbiont of Oedothorax gibbosus
Wolbachia endosymbiont (group A) of Crataerina pallida
Wolbachia endosymbiont of Listronotus oregonensis
Wolbachia endosymbiont of Delia radicum
Candidatus Lariskella endosymbiont of Epinotia ramella
Candidatus Tisiphia endosymbiont of Beris chalybata
Wolbachia endosymbiont (group B) of Dendrolimus pini
Wolbachia endosymbiont (group B) of Gerris lacustris
Wolbachia endosymbiont of Diaphorina citri
Wolbachia endosymbiont (group A) of Limnophora tigrina
Wolbachia endosymbiont (group B) of Catoptria pinella
Wolbachia endosymbiont (group A) of Udea olivalis
Candidatus Trichorickettsia mobilis
Wolbachia endosymbiont (group B) of Cyclophora punctaria
Wolbachia endosymbiont (group B) of Aricia artaxerxes
Wolbachia endosymbiont (group A) of Endotricha flammealis
Wolbachia pipientis
Candidatus Tisiphia endosymbiont of Oplodontha viridula
Wolbachia endosymbiont of Oryzaephilus surinamensis_JKI−2014
Rickettsiales endosymbiont of Stachyamoeba lipophora_RICK01
Wolbachia endosymbiont (group B) of Endotricha flammealis
Wolbachia endosymbiont (group B) of Chorthippus parallelus
Rickettsia endosymbiont of Pantilius tunicatus
Wolbachia endosymbiont (group A) of Clivina fossor
Wolbachia endosymbiont (group B) of Rhopobota naevana
Rickettsia tamurae subsp. buchneri
Wolbachia endosymbiont (group A) of Epagoge grotiana
Wolbachia endosymbiont (group B) of Idaea biselata
Wolbachia endosymbiont of Phyllotreta cruciferae
Wolbachia endosymbiont of Folsomia candida_Berlin
Wolbachia endosymbiont (group B) of Euphydryas aurinia
Wolbachia endosymbiont (group A) of Philonthus cognatus
Rickettsia endosymbiont of Ceutorhynchus obstrictus
Candidatus Tisiphia endosymbiont of Thecophora atra
Wolbachia endosymbiont (group B) of Melanostoma mellinum
Rickettsia endosymbiont of Gonocerus acuteangulatus
Candidatus Tisiphia endosymbiont of Ditula angustiorana
Rickettsia endosymbiont of Polydrusus tereticollis
Wolbachia endosymbiont (group B) of Ischnura elegans
Candidatus Tisiphia endosymbiont of Parasteatoda lunata
Orientia tsutsugamushi str. Gilliam
Rickettsia endosymbiont of Oedothorax gibbosus
Rickettsia sp. str. rMo
0e+00
1e+06
2e+06
3e+06
genome_size

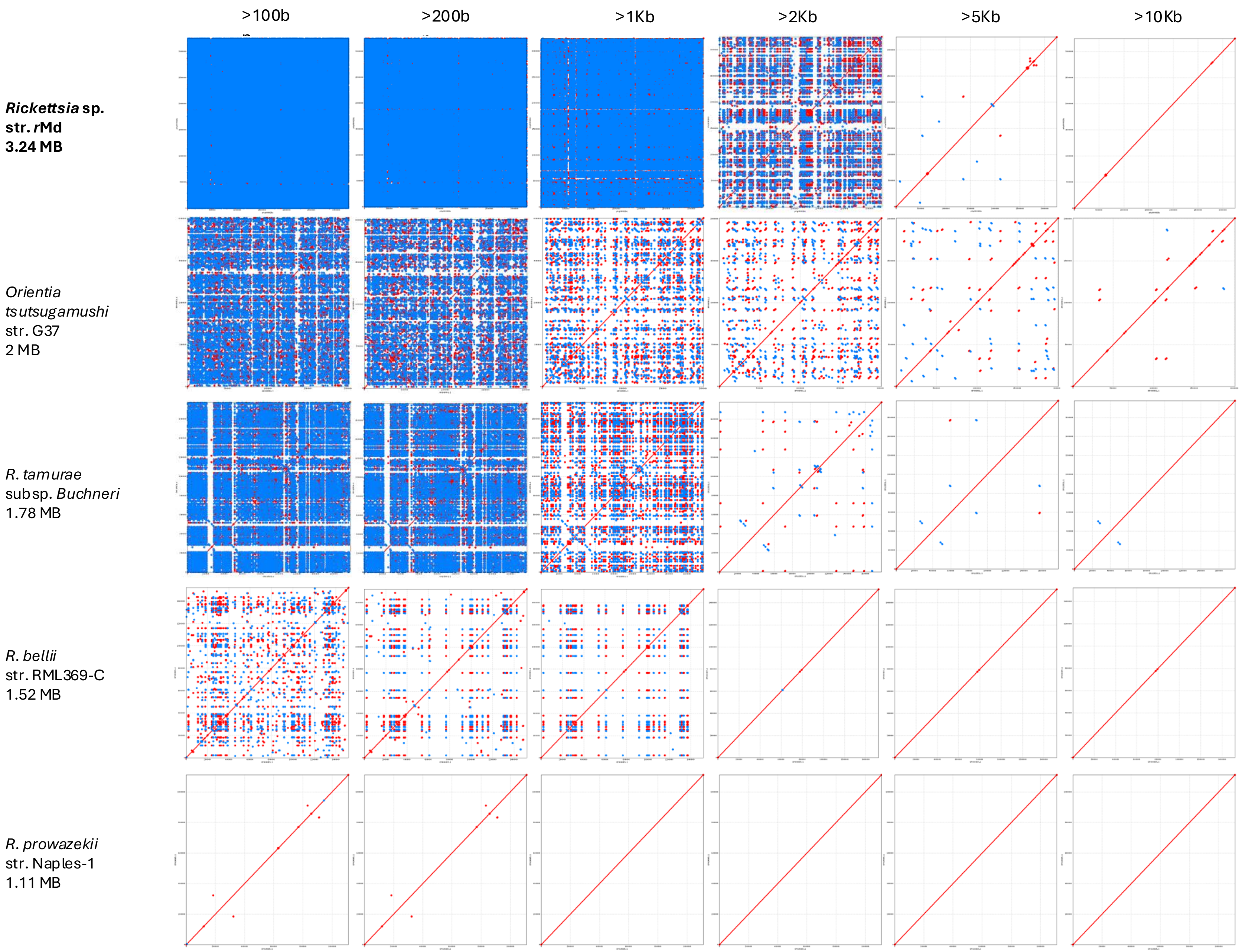


**Figure S4**. Mummerplots depicting repetitive sequences in the main chromosomes of *Rickettsia r*Md (**bold**) and four others of varying genome size. Repeats were filtered by size then plotted. For each graph, the X-axis and the Y-axis are the same; i.e. the main chromosome is plotted against itself. Repeats are shown in blue (forward direction), and red (reverse direction).

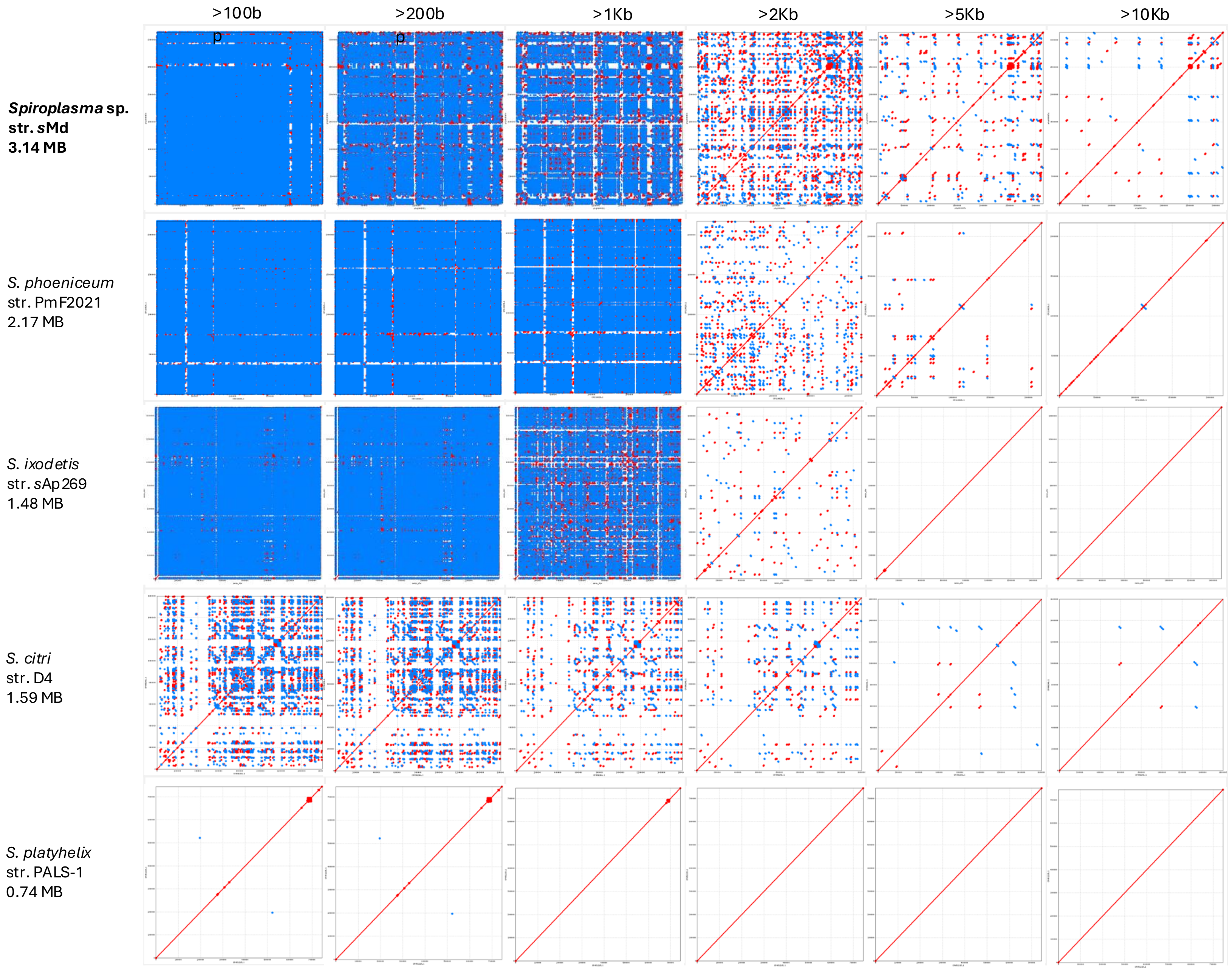


**Figure S5** Mummerplots depicting repetitive sequences in the main chromosomes of *Spiroplasma* sMd (**bold**) and four others of varying genome size. Repeats were filtered by size then plotted. For each graph, the X-axis and the Y-axis are the same; i.e. the main chromosome is plotted against itself. Repeats are shown in blue (forward direction), and red (reverse direction).

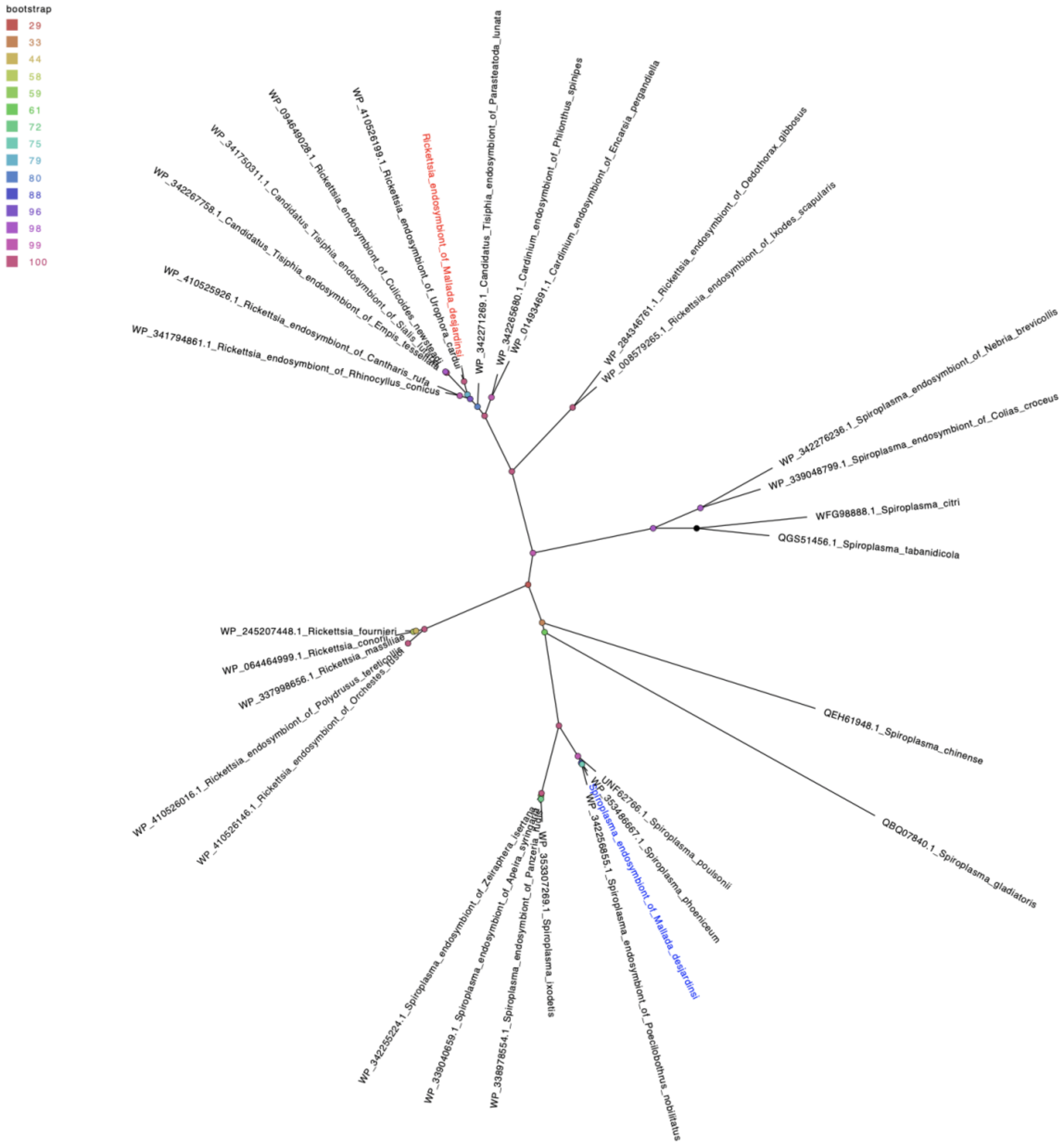


**Figure S6**. Star phylogeny (best-fit protein model according to Bayesian Information Criterion - VT+F+G4) of IS3 family transposase amino acid sequences from *Spiroplasma* sMd (in blue), *Rickettsia r*MD (in red) alongside selected strains from both *Rickettsia* and *Spiroplasma*.

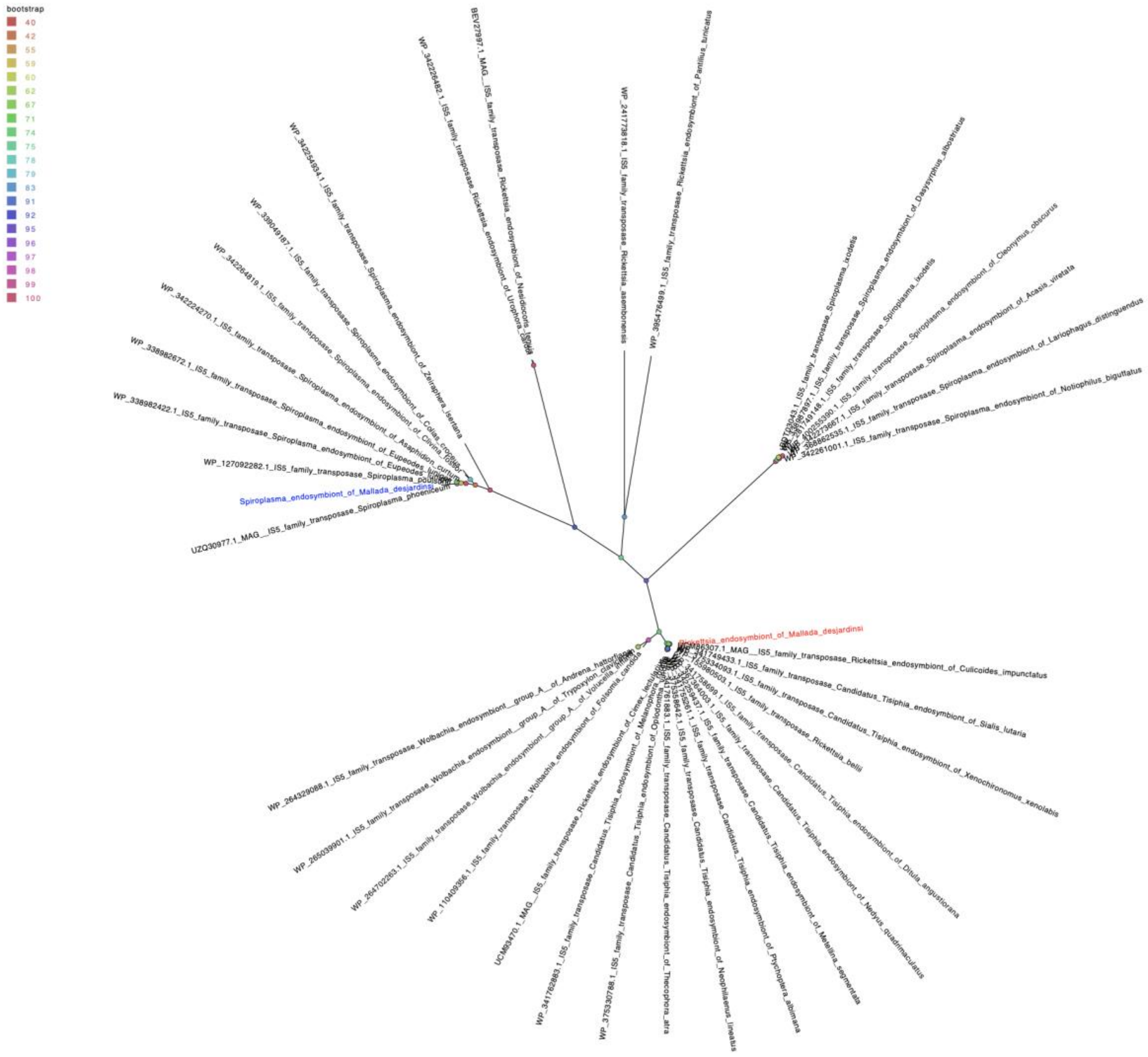


**Figure S7**. Star phylogeny (best-fit protein model according to Bayesian Information Criterion - JTT+F+G4) of IS5 family transposase amino acid sequences from *Spiroplasma s*Md (in blue), *Rickettsia r*MD (in red) alongside selected strains from both *Rickettsia* and *Spiroplasma*.

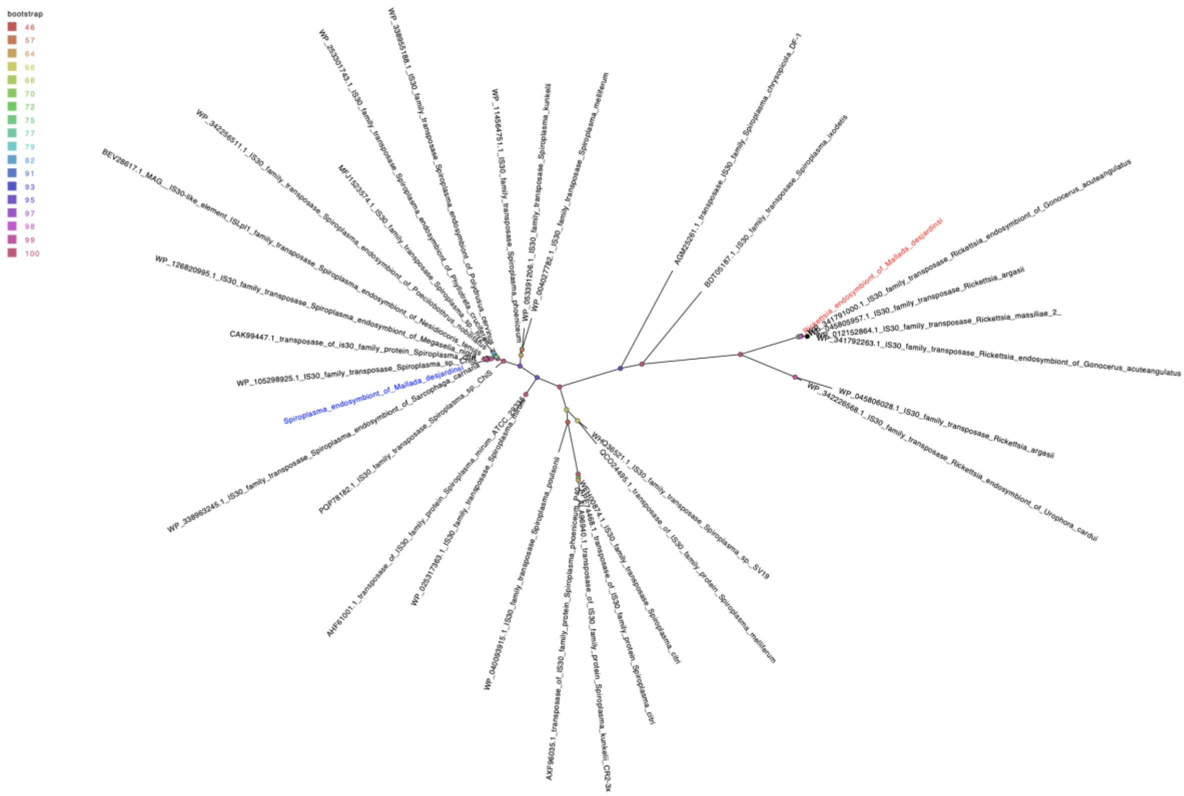

**Figure S8**. Star phylogeny (best-fit protein model according to Bayesian Information Criterion - VT+F+I+G4) of IS30 family transposase amino acid sequences from *Spiroplasma s*Md (in blue), *Rickettsia r*MD (in red) alongside selected strains from both *Rickettsia* and *Spiroplasma*.

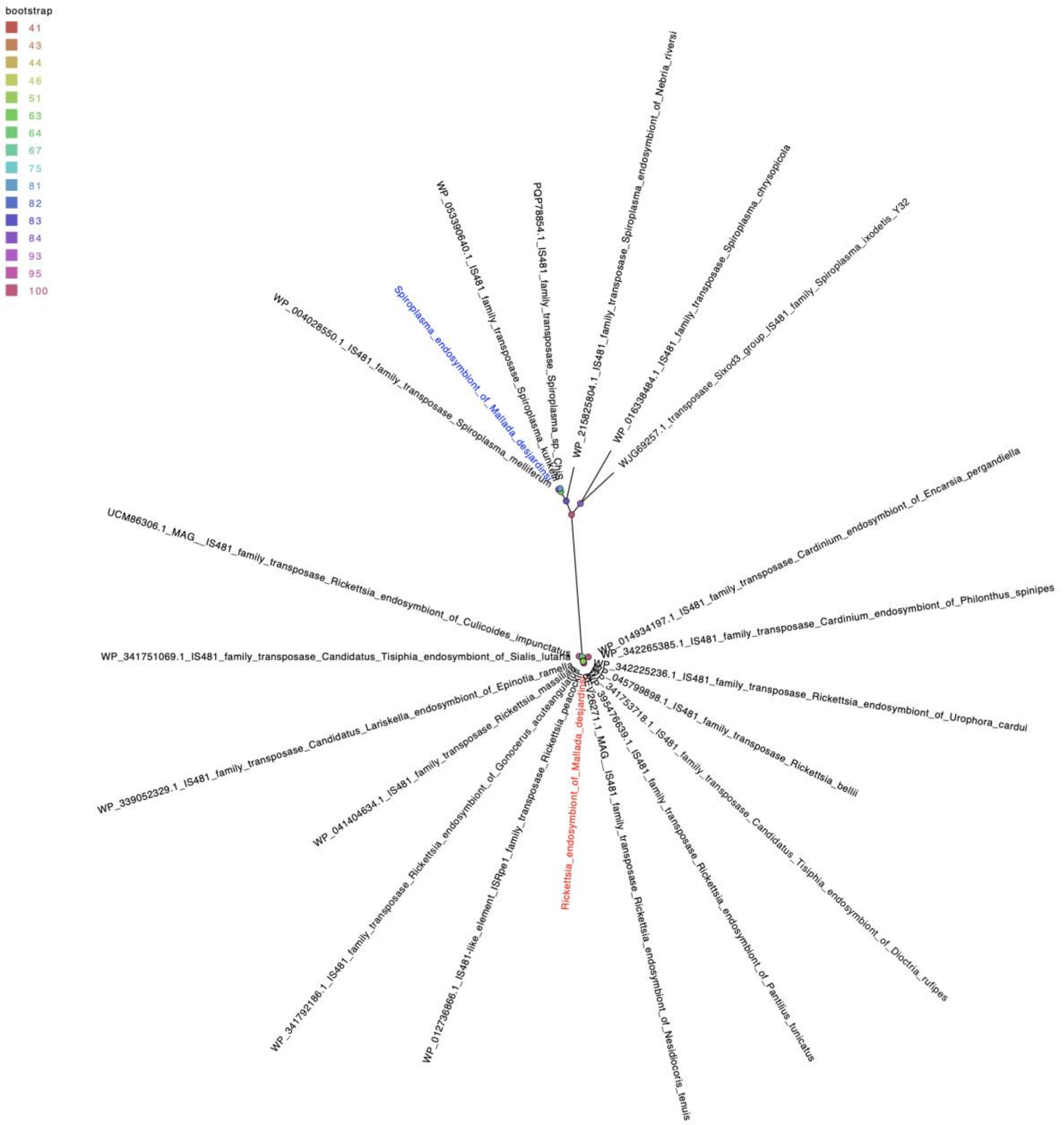


**Figure S9**. Star phylogeny (best-fit protein model according to Bayesian Information Criterion - Q.pfam+F+R3) of IS481 family transposase amino acid sequences from *Spiroplasma s*Md (in blue), *Rickettsia r*MD (in red) alongside selected strains from both *Rickettsia* and *Spiroplasma*.

**Figure S10** *Rickettsia* kegg module analysis

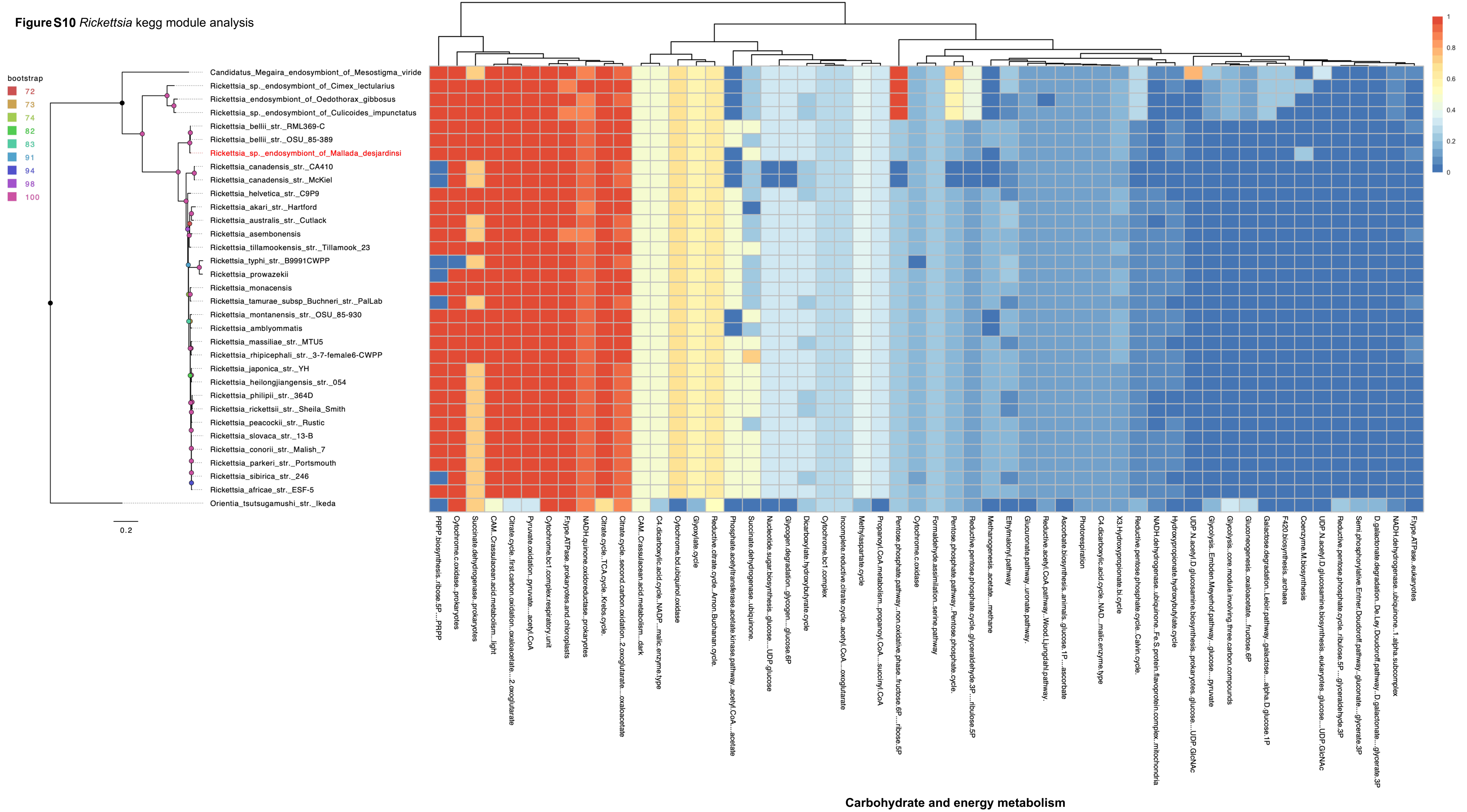

**Figure S11** *Rickettsia* kegg module analysis

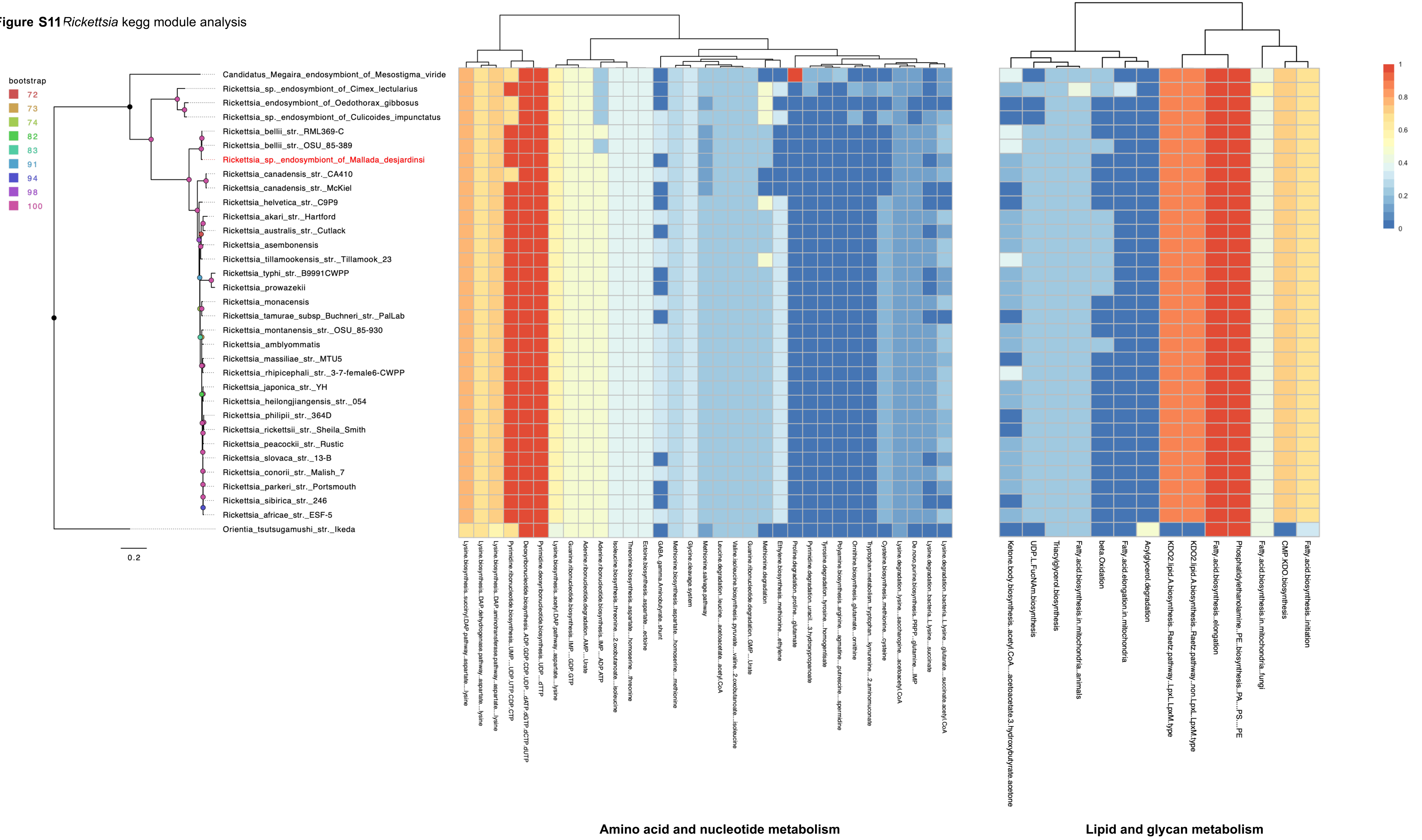

**Figure S12** *Rickettsia* kegg module analysis

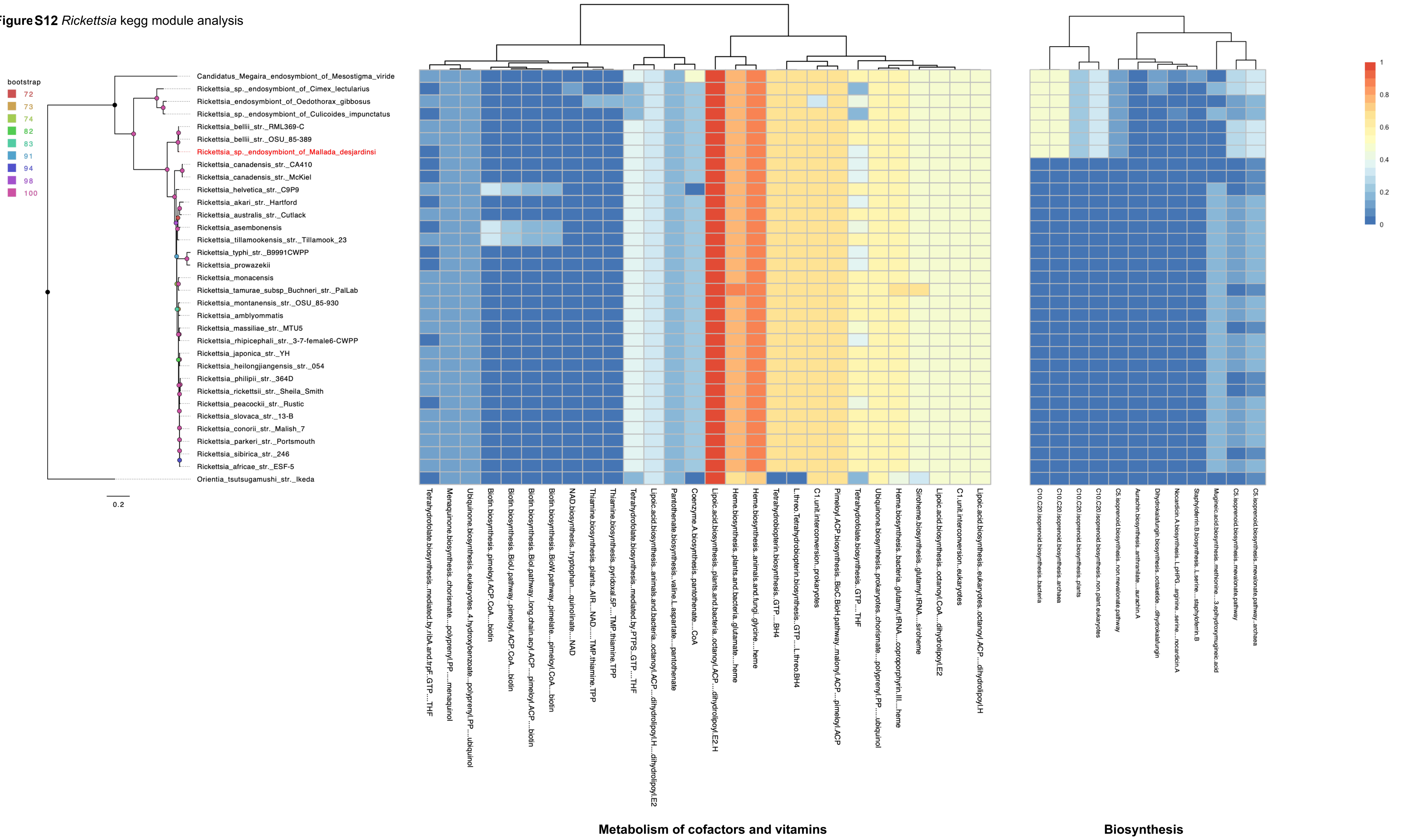

**Figure S13** *Spiroplasma* kegg module analysis

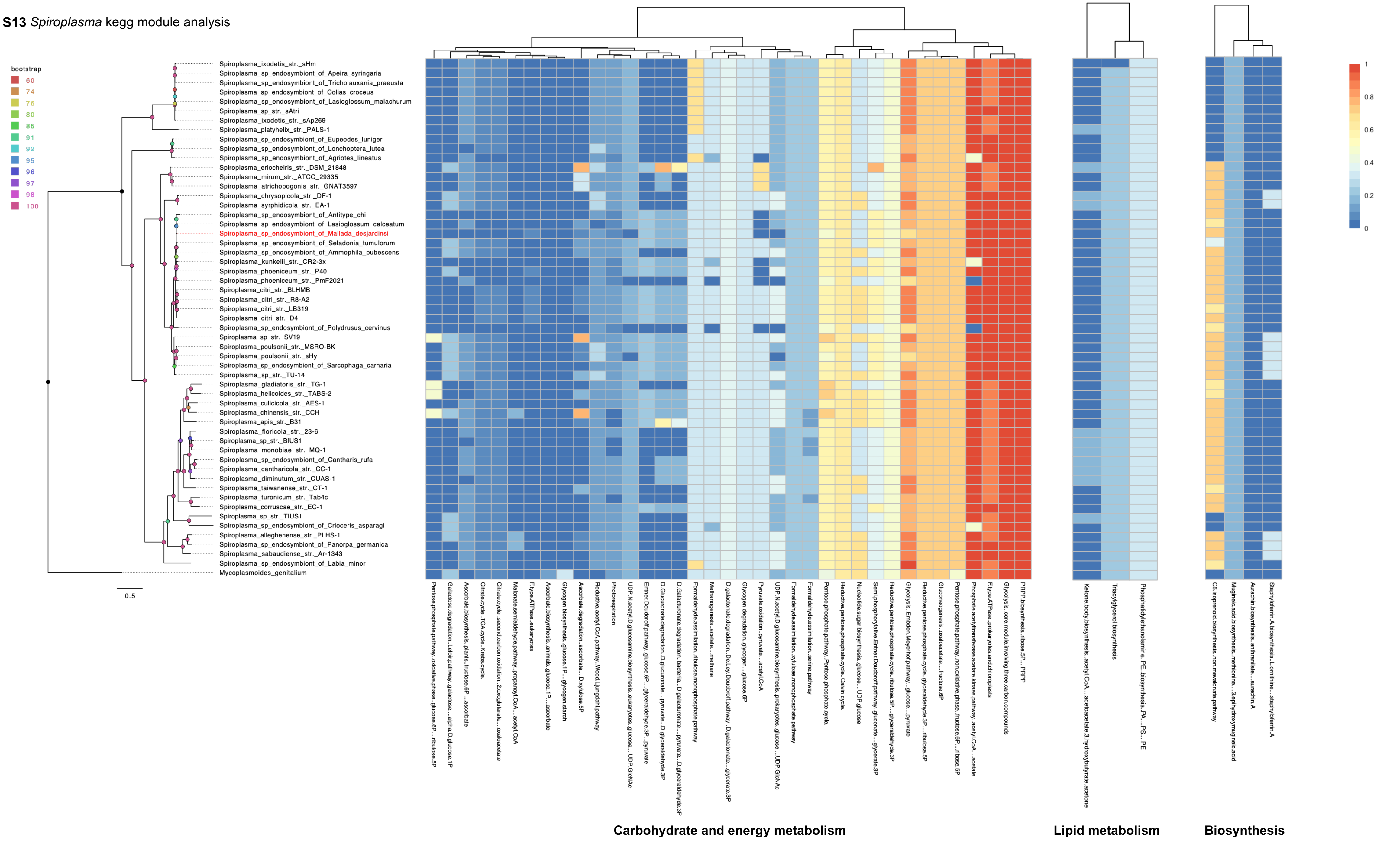

**Figure S14** *Spiroplasma* kegg module analysis

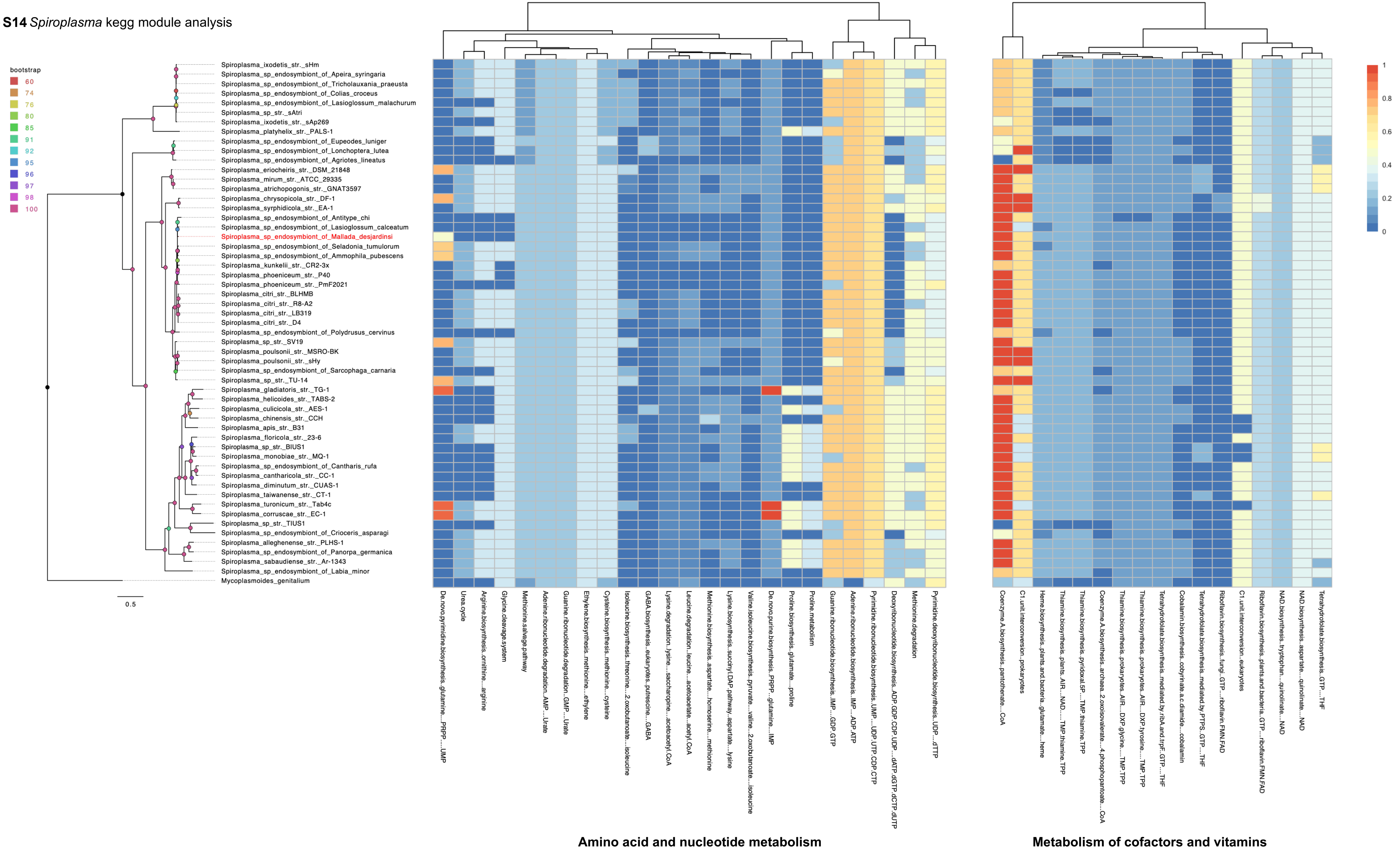

Figure legends for Figures S2, S3, S10-S14

Figure S2. Genome sizes of sequenced complete Mollicute bacteria. *Spiroplasma* sp. str. *s*Md is highlighted in yellow.

Figure S3. Genome sizes of sequenced complete Rickettsiales bacteria. *Rickettsia* sp. str. *r*Md is highlighted in yellow.

Figure S10. *Rickettsia* kegg module analysis – carbohydrate and energy metabolism

Figure S11. *Rickettsia* kegg module analysis – amino acid, nucleotide, lipid and glycan metabolism

Figure S12. *Rickettsia* kegg module analysis – metabolism of cofactors and vitamins, and biosynthesis

Figure S13. *Spiroplasma* kegg module analysis – carbohydrate and energy metabolism, lipid metabolism and biosynthesis

Figure S14. *Spiroplasma* kegg module analysis – amino acid and nucleotide metabolism, and metabolism of cofactors and vitamins
